# On Double Cyclic Codes over Finite Chain Rings for DNA Computing

**DOI:** 10.3390/e27121187

**Published:** 2025-11-22

**Authors:** Shakir Ali, Amal S. Alali, Mohd Azeem, Atif Ahmad Khan, Kok Bin Wong

**Affiliations:** 1 Department of Mathematics, Faculty of Science, Aligarh Muslim University, Aligarh 202002, India; mohdazeem9211@gmail.com (M.A.); atifkhanalig1997@gmail.com (A.A.K.); 2 Institute of Mathematical Sciences, Faculty of Science, Universiti Malaya, Kuala Lumpur 50603, Malaysia; kbwong@um.edu.my; 3 Department of Mathematical Sciences, College of Science, Princess Nourah bint Abdulrahman University, P.O. Box 84428, Riyadh 11671, Saudi Arabia; asalali@pnu.edu.sa

**Keywords:** double cyclic code, chain ring, DNA code, reversible code, reversible-complement code, 94B05, 94B15, 94B60

## Abstract

Let *e* be a fixed positive integer and $n_{1},n_{2}$ be odd positive integers. The main objective of this article is to investigate the algebraic structure of double cyclic codes of length $\left(n_{1},n_{2}\right)$ over the finite chain ring Re = F4e+vF4e, where $v^{2}=0$. Building upon this structural framework, we further demonstrate the construction of DNA codes derived from these double cyclic codes over Re. In addition, we provide the necessary and sufficient criteria showing that these codes possess reversibility and reverse-complement properties over Re. Furthermore, we introduce a generalized Gray map that extends the classical Gray map from the ring $F_{2}+vF_{2}$ with $v^{2}=0$ to the ring Re, showing a direct correspondence between elements of Re and DNA sequences over S={A,T,G,C} utilizing double cyclic codes. To illustrate the applicability of our results, we present some examples demonstrating the effectiveness of the mapping in generating reversible and reverse-complement DNA codes from algebraic structures over the ring Re.

## 1. Introduction

Deoxyribonucleic acid (DNA) serves as the genetic material in nearly all plant and animal species, whereas some viruses utilize either DNA or RNA instead. The transmission of hereditary information across generations occurs through the mechanism of DNA replication. Furthermore, as demonstrated by DNA fingerprinting methods, DNA is essential for both recognizing individual traits and comprehending the evolutionary links across species. Each of the two strands that make up DNA’s double helix shape is made up of nucleotide polymers. A deoxyribose sugar, a phosphate group, and a nitrogenous base such as adenine (A), thymine (T), cytosine (C), or guanine (G) are components of every nucleotide. The 5′ and 3′ ends of the strands are polar terminals that are chemically different. The Watson–Crick complement (WCC) rule states that adenine pairs with thymine and cytosine pairs with guanine for the bases of the two DNA strands. To indicate that $\bar{A}=T$, $\bar{T}=A$, $\bar{G}=C$, and $\bar{C}=G$, we use $\bar{x}$ to represent the complement of a base *x*. The DNA strand $5^{\prime }-\mathrm{ACTGGCA}-3^{\prime }$, for instance, is coupled with the strand $3^{\prime }-\mathrm{TGACCGT}-5^{\prime }$, which, after reorientation, may also be expressed as $5^{\prime }-\mathrm{TGCCAGT}-3^{\prime }$. Adleman [1] conducted a pioneering experiment in DNA computing in 1994, showing how to solve a seven-node directed Hamiltonian route problem using DNA molecules. Many scholars have since built on this work and used DNA computing approaches to solve a range of mathematical problems [2,3,4,5,6,7,8]. In the context of designing DNA codes, a number of crucial limitations have been found to demonstrate the codes’ dependability and biological viability. These consist of the constant GC-content constraint, the reverse constraint, the reverse-complement constraint, and the Hamming constraint for a minimum distance *d*. The reverse of *x* is defined as $x^{r}=\left(x_{n},x_{n-1},\ldots ,x_{1}\right)$ for each codeword $x=(x_{1},x_{2},\ldots ,x_{n})$, where $x_{i}\in \left\{A,T,G,C\right\}$, the complement is defined as $\bar{x}=\left(\bar{x_{1}},\bar{x_{2}},\ldots ,\bar{x_{n}}\right)$, where $\bar{x_{i}}$ represents the Watson–Crick complement, and $x^{rc}=\bar{x^{r}}=\left(\bar{x_{n}},\bar{x_{n-1}},\ldots ,\bar{x_{1}}\right)$ for the reverse-complement. The Hamming distance, represented by $d_{H}\left(x,y\right)$, between two codewords, *x* and *y*, is the number of places at which the respective symbols differ.

Early studies of DNA code design focused on the structural similarities between four-element algebraic systems and the DNA alphabet. As a result, early research concentrated on sets of four elements that had an algebraic structure. In [9], Gaborit and King investigated approaches for constructing DNA codes through additive and linear codes defined on the four-element finite field. Four combinatorial restrictions that define a high-quality DNA code were suggested by them. We follow the definition of a DNA code from [10]. A code is said to be a DNA code $C_{DNA}$ if it satisfies at least one of the following constraints:

(i) **Hamming distance constraint:** For a given minimum distance *d*, $d_{H}\left(x,y\right)\geq d$ for each $x,y\in C_{DNA}$ with x≠y.
(ii) **Reverse constraint:** For any $x,y\in C_{DNA}$, including x=y, has $d_{H}\left(x,y^{r}\right)\geq d$.
(iii) **Reverse-complement constraint:** For any $x,y\in C_{DNA}$, $d_{H}\left(x,y^{rc}\right)\geq d$, including x=y.
(iv) **Fixed GC-content constraint:** The number of guanine (G) and cytosine (C) bases in each codeword $x\in C_{DNA}$ must be equal.

The design of linear and cyclic codes of odd length over $F_{4}$ that are appropriate for DNA computing was examined by Abualrub et al. [11]. Since then, there has been a lot of interest in the research of cyclic codes in relation to DNA computing. Cyclic DNA codes over the finite ring $F_{2}+uF_{2}$ with $u^{2}=1$ were studied by Siap et al. [12]. Subsequently, Guenda and Gulliver [13] concentrated on odd-length cyclic codes over the finite ring $F_{2}+uF_{2}$ with $u^{2}=0$. Four elements make up this finite commutative chain ring: 0, 1, *u*, and 1+u. Its maximal ideal 〈u〉 and characteristic is 2. The DNA bases and the ring elements were found to match one-to-one in the following manner: 0↦A, 1↦G, u↦T, and 1+u↦C. They constructed cyclic codes that met the GC-content and reverse-complement constraints using this mapping. This study was further expanded to include cyclic DNA codes of even length across the same ring by Liang and Wang [10], who focused on the prerequisites for reversibility and reverse-complement limitations. A few years later, the scope of DNA code investigations in coding theory was extended to more complex algebraic systems, such as finite rings like $F_{4}+vF_{4}$ with $v^{2}=v$, $F_{2}+uF_{2}+vF_{2}+uvF_{2}$ with $u^{2}=0,v^{2}=v,uv=vu$, and $F_{2}\left[u\right]/\langle u^{4}-1\rangle$, (see Bayram et al. [14], Zhu and Chen [15], Yildiz and Siap [16], respectively). Subsequent research has focused on examining cyclic DNA codes constructed over various finite rings by Liu and Liu [17] and Prakash et al. [18]. In recent years, Dinh et al. [19] presented $F_{4}RS$-cyclic codes of block length (α,β,γ), a unique method for creating DNA codes by investigating cyclic codes over mixed alphabets. In this case, $S=F_{4}+uF_{4}+vF_{4}$ with $u^{2}=u$, $v^{2}=v$, uv=vu=0, and $R=F_{4}+uF_{4}$ with $u^{2}=u$. These codes can be viewed as S[x]-submodules of $F_{4}\left[x\right]/\langle x^{\alpha }-1\rangle \times R\left[x\right]/\langle x^{\beta }-1\rangle \times S\left[x\right]/\langle x^{\gamma }-1\rangle$. They determined the circumstances in which separable cyclic codes meet the reversible and reverse-complement constraints and investigated the structural characteristics of these codes over $F_{4}RS$.

In this study, we use double cyclic codes, a new kind of linear code, to study DNA codes. According to research by Dinh et al. [20], these codes are comparable to cyclic codes over mixed alphabets. Recent developments on mixed-ring and double-circulant constructions further support the relevance of our approach. Shi et al. [21] analyzed structural properties of $Z_{2}Z_{4}$-additive quasi-cyclic codes, while Wang and Shi [22] investigated Gray images of cyclic codes over $Z_{p^{2}}$ and $Z_{p}Z_{p^{2}}$. In addition, Shi et al. [23] established bounds on the minimum distance of double-circulant cubic residue codes, emphasizing the significance of $Z_{p}Z_{p^{2}}$-linear and double-circulant structures. However, our focus is on codes of block length $\left(n_{1},n_{2}\right)$ across a single alphabet. Codewords that may be divided into two pieces so that a simultaneous cyclic shift of both portions yields another codeword inside the same code are known as double cyclic codes. In the investigation of double cyclic codes of length (α,β) over $Z_{2}$, attention was given to their algebraic structure and generator polynomials; Borges et al. [24] developed the idea of double cyclic codes. According to earlier research by Siap and Kulhan [25], these codes are closely connected to generalized quasi-cyclic codes of index 2. Double cyclic codes of length (α,β) over $Z_{2}$ were demonstrated to represent $Z_{2}\left[x\right]$-submodules of the module $Z_{2}\left[x\right]/\langle x^{\alpha }-1\rangle \times Z_{2}\left[x\right]/\langle x^{\beta }-1\rangle$. The algebraic structure and duality features of double cyclic codes over other finite rings were investigated by a number of researchers after this influential work (see [26,27,28,29] for more details). Additionally, double cyclic codes over $Z_{4}$ were shown to be asymptotically excellent by Gao and Hou [30], indicating their potential for real-world uses in coding theory and DNA-based data storage. In recent work, Kanlaya and Klin-Eam [31] investigated double cyclic codes defined over $F_{2}+uF_{2}$ with the condition $u^{2}=0$, focusing on applications in DNA coding. Motivated by constructions involving cyclic codes over finite rings and mixed alphabets, we extend this line of research by developing double cyclic codes of length $\left(n_{1},n_{2}\right)$ over the finite chain rings Re=F4e+vF4e, where $v^{2}=0$, demonstrating their relevance to DNA codes.

The structure of the paper is outlined as follows: Double cyclic codes of length $\left(n_{1},n_{2}\right)$ over the finite chain ring Re=F4e+vF4e, where $v^{2}=0$, are studied in Section 2. The construction of double cyclic codes appropriate for DNA coding is the main motive of Section 3. There are three subsections in this section. The necessary and sufficient conditions for a double cyclic code to be reversible and reversible-complement are established in the first and second subsections, respectively, while in the third subsection, we introduce a generalized Gray map that extends the classical Gray map from the ring $F_{2}+vF_{2}$ with $v^{2}=0$ to Re along with examples to validate the theoretical findings. In Section 4, we explore applications to DNA data storage, DNA-based authentication, and cryptographic systems, illustrating the practical effectiveness of the reversible DNA codes developed through the framework of double cyclic codes. Section 5 concludes the paper with a summary of our results and directions for future research.

## 2. Preliminaries

Let *e* be a fixed positive integer and $n_{1},n_{2}$ be odd positive integers such that $n=n_{1}+n_{2}$. In this research, we describe the ring Re=F4e+vF4e with $v^{2}=0$ using the notation Re. The ring Re represents a finite commutative chain ring with characteristic 2 whose maximal ideal is 〈v〉. A linear code of length *n* over Re is defined as a subset C⊆Rne that forms an Re-submodule of Rne. For any $(a_{0},a_{1},\ldots ,a_{n-1})\in C$, the tuple $(a_{n-1},a_{0},\ldots ,a_{n-2})\in C$ is closed, indicating that a linear code *C* is cyclic. The *n* coordinate set is divided into two subsets, representing $n_{1}$ and $n_{2}$ coordinates, respectively. Therefore, every subset *C* of Rne may be thought of as a subset of the Cartesian product Rn1e×Rn2e, which we represent by Rn1,n2e. According to the framework presented by Borges et al. [24], for any k∈Rne and u=(a0,a1,…,an1−1;b0,b1,…,bn2−1)∈Rn1,n2e, we define the scalar multiplication of *k* with u as follows:

$$k*\left(a_{0},a_{1},\ldots ,a_{n_{1}-1};b_{0},b_{1},\ldots ,b_{n_{2}-1}\right)=\left(ka_{0},ka_{1},\ldots ,ka_{n_{1}-1};kb_{0},kb_{1},\ldots ,kb_{n_{2}-1}\right).$$

Using the multiplication ∗, this construction implies that Rn1,n2e forms an Re-module. Consequently, a linear code *C* of length *n* over the ring Re can be characterized as an Re-submodule of Rn1,n2e. For convenience, we denote any element u=(a0,a1,…,an1−1;b0,b1,…,bn2−1)∈Rn1,n2e simply as u=(a;b), where $a=(a_{0},a_{1},\ldots ,a_{n_{1}-1})$ and $b=(b_{0},b_{1},\ldots ,b_{n_{2}-1})$. In analogy with the notion of double cyclic codes over finite fields and rings (see [24,27,28,29] for further details), we present the definition of double cyclic codes over Re as follows:

**Definition** **1.**  *Let C be a linear code of length $\alpha =\alpha_{1}+\alpha_{2}$ over Re, where $\alpha_{1},\alpha_{2}$ be non-negative integers. The code C is called a double cyclic code of length $\left(\alpha_{1},\alpha_{2}\right)$ if*

$$(a_{\alpha_{1}-1},a_{0},a_{1},\ldots ,a_{\alpha_{1}-2};b_{\alpha_{2}-1},b_{0},b_{1},\ldots ,b_{\alpha_{2}-2})\in C$$

*for all*

$$(a_{0},a_{1},\ldots ,a_{\alpha_{1}-1};b_{0},b_{1},\ldots ,b_{\alpha_{2}-1})\in C.$$

*The code C is a cyclic code of length $\alpha_{2}$ over Re if $\alpha_{1}=0$. Analogously, C is called a cyclic code of length $\alpha_{1}$ over Re if $\alpha_{2}=0$. The code C is referred to as a separable double cyclic code of length $\left(\alpha_{1},\alpha_{2}\right)$ over Re if it can be expressed as $C=C_{\alpha_{1}}\times C_{\alpha_{2}}$, where $C_{\alpha_{1}}$ and $C_{\alpha_{2}}$ denote the coordinate projections of C onto the first $\alpha_{1}$ and the last $\alpha_{2}$ coordinates, respectively. A code that is not separable refers to a non-separable code.*

**Example** **1.**  *Let $C=\langle \left(v\left(1+x+x^{2}\right),0\right),\left(1+v,v\left(1+x+x^{2}\right)\left(1+x^{3}+x^{6}\right)\right)\rangle$ be a double cyclic code of length (3,9) over R1 (for e=1). It is straightforward to see that C is non-separable code over R1.*

*We denote Re[x]〈xn1−1〉×Re[x]〈xn2−1〉 by Rn1,n2e, and the image of the vector (a;b) by (a(x);b(x)). Now define a bijective map ζ as follows:*

ζ:Rn1,n2e→Rn1,n2e by


$$\zeta \left(a_{0},a_{1},\ldots ,a_{n_{1}-1};b_{0},b_{1},\ldots ,b_{n_{2}-1}\right)=\left(a_{0}+a_{1}x+\ldots +a_{n_{1}-1}x^{n_{1}-1};b_{0}+b_{1}x+\ldots +b_{n_{2}-1}x^{n_{2}-1}\right).$$

*Let k(x)∈Re[x] and u(x)=(a(x);b(x))∈Rn1,n2e. The multiplication between k(x) and u(x) is defined by*

$$k\left(x\right)*\left(a\left(x\right);b\left(x\right)\right)=\left(k\left(x\right)a\left(x\right)\;mod\;\left(x^{n_{1}}-1\right);k\left(x\right)b\left(x\right)\;mod\;\left(x^{n_{2}}-1\right)\right).$$

*With this operation, Rn1,n2e becomes an Re[x]-module under the multiplication ∗. For convenience, throughout this article, we replace $\left(k\left(x\right)a\left(x\right)\;mod\;\left(x^{n_{1}}-1\right);k\left(x\right)b\left(x\right)\;mod\;\left(x^{n_{2}}-1\right)\right)$ with (k(x)a(x);k(x)b(x)).*

*Now, consider an element u(x)=(a(x);b(x))=(a0+a1x+…+an1−1xn1−1;b0+b1x+…+bn2−1xn2−1)∈Rn1,n2e. Then, the multiplication of u(x) by x under ∗ is given by*

$$x*\left(a\left(x\right);b\left(x\right)\right)=\left(a_{n_{1}-1}+a_{0}x+a_{1}x^{2}+\ldots +a_{n_{1}-2}x^{n_{1}-1};b_{n_{2}-1}+b_{0}x+b_{1}x^{2}+\ldots +b_{n_{2}-2}x^{n_{2}-1}\right).$$

*This demonstrates that multiplying u(x) by x corresponds to a cyclic shift of its components. Hence, the following proposition states that a double cyclic code of length $\left(n_{1},n_{2}\right)$ over Re can be identified as an Re[x]-submodule of Rn1,n2e:*

**Proposition** **1.**  *Let C be a subset of Rn1,n2e. Then, C is a double cyclic code of length $\left(n_{1},n_{2}\right)$ over the ring Re iff ζ(C) is an Re[x]-submodule of Rn1,n2e.*

**Proof.**  Let *C* be a double cyclic code of length $\left(n_{1},n_{2}\right)$ over Re. Then, *C* is an Re-submodule of Rn1,n2e, and consequently, ζ(C) forms a subgroup of Rn1,n2e. For any element $(a_{0},a_{1},\ldots ,a_{n_{1}-1};b_{0},b_{1},\ldots ,b_{n_{2}-1})\in C$, the corresponding polynomial $u\left(x\right)=\left(a\left(x\right);b\left(x\right)\right)=\left(a_{0}+a_{1}x+\ldots +a_{n_{1}-1}x^{n_{1}-1};b_{0}+b_{1}x+\ldots +b_{n_{2}-1}x^{n_{2}-1}\right)=\zeta \left(a_{0},a_{1},\ldots ,a_{n_{1}-1};b_{0},b_{1},\ldots ,b_{n_{2}-1}\right)\;\mathrm{of}\;\zeta (C)$ satisfies x∗u(x)∈ζ(C), since *C* is a double cyclic code. Therefore, applying the operation again yields $x^{2}*u\left(x\right)=x*\left(x*u\left(x\right)\right)\in \zeta \left(C\right)$. By induction, it follows that $x^{i}*u\left(x\right)\in \zeta \left(C\right)$ for all i≥0.

Let r(x)=r0+r1x+…+rtxt∈Re[x] be any polynomial with non-negative integer *t*. Then, we have

$$r\left(x\right)*u\left(x\right)=\left(r_{0}+r_{1}x+\ldots +r_{t}x^{t}\right)*u\left(x\right)=\left(r_{0}*u\left(x\right)\right)+\left(r_{1}x*u\left(x\right)\right)+\ldots +\left(r_{t}x^{t}*u\left(x\right)\right).$$

This implies that r(x)∗u(x)∈ζ(C) for any u(x)∈ζ(C) and r(x)∈Re[x]. Hence, ζ(C) is an Re[x]-submodule of Rn1,n2e.

Conversely, suppose that ζ(C) is an Re[x]-submodule of Rn1,n2e. Then, *C* is an Re-submodule of Rn1,n2e. Let $u=(a_{0},a_{1},\ldots ,a_{n_{1}-1};b_{0},b_{1},\ldots ,b_{n_{2}-1})$ be a codeword in *C*. The corresponding polynomial representation is given by $u\left(x\right)=\zeta \left(u\right)=\left(a_{0}+a_{1}x+\ldots +a_{n_{1}-1}x^{n_{1}-1};b_{0}+b_{1}x+\ldots +b_{n_{2}-1}x^{n_{2}-1}\right)$. By assumption, x∗u(x)∈ζ(C), which implies that $(a_{n_{1}-1},a_{0},\ldots ,a_{n_{1}-2};b_{n_{2}-1},b_{0},\ldots ,b_{n_{2}-2})\in C.$ Therefore, *C* is a double cyclic code of length $\left(n_{1},n_{2}\right)$ over Re. □

In view of Proposition 1, a double cyclic code *C* having length $\left(n_{1},n_{2}\right)$ over the ring Re can be viewed as an Re[x]-submodule of Rn1,n2e. After this, we substitute *C* for ζ(C) in the double cyclic code *C* to make things simpler.

Throughout the entire article, two odd positive integers, $n_{1}$ and $n_{2}$, are taken into consideration so that $n=n_{1}+n_{2}$. Consider *C* to be an Re[x]-submodule of Rn1,n2e. Define the following Re[x]-module homomorphisms as ζn1:C→Re[x]〈xn1−1〉 = Rn1e by

$$\zeta_{n_{1}}\left(a\left(x\right);b\left(x\right)\right)=a\left(x\right)$$

for each (a(x);b(x))∈C⊆Rn1,n2e, and define ζn2:C→Re[x]〈xn2−1〉=Rn2e by

$$\zeta_{n_{2}}\left(a\left(x\right);b\left(x\right)\right)=b\left(x\right)$$

for each (a(x);b(x))∈C⊆Rn1,n2e. Then, both $\zeta_{n_{1}}$ and $\zeta_{n_{2}}$ are Re[x]-module homomorphisms. Moreover, if $\zeta_{n_{1}}\left(C\right)$ is an ideal of Rn1e, then from the study of Liang and Wang [10], we have

$$\zeta_{n_{1}}\left(C\right)=\langle f_{0}\left(x\right),vf_{1}\left(x\right)\rangle =\langle f_{0}\left(x\right)+vf_{1}\left(x\right)\rangle ,$$

where $f_{0}\left(x\right),f_{1}\left(x\right)\in F_{4^{e}}\left[x\right]$ satisfy $f_{1}\left(x\right)|f_{0}\left(x\right)|\left(x^{n_{1}}-1\right)$. Similarly, if $\zeta_{n_{2}}\left(C\right)$ is an ideal of Rn2e, then

$$\zeta_{n_{2}}\left(C\right)=\langle g_{0}\left(x\right)+vg_{1}\left(x\right)\rangle ,$$

with $g_{0}\left(x\right),g_{1}\left(x\right)\in F_{4^{e}}\left[x\right]$ satisfying $g_{1}\left(x\right)|g_{0}\left(x\right)|\left(x^{n_{2}}-1\right)$. Next, we examine the generator polynomials associated with a double cyclic code *C* of length $\left(n_{1},n_{2}\right)$ over the ring Re. For f(x)∈Re[x] and a fixed positive integer *n*, one can find polynomials q(x) and r(x) in Re[x] satisfying $f\left(x\right)=\left(x^{n}-1\right)q\left(x\right)+r\left(x\right),$ with the condition deg(r(x))≤n−1. We denote the remainder r(x) by ${\left[f\left(x\right)\right]}_{\left(x^{n}-1\right)}$.

By adopting the same procedures as in Gao et al. [28], the subsequent theorem and lemma are established.

**Theorem** **1.**  *Let C be a double cyclic code of length $\left(n_{1},n_{2}\right)$ over Re. Then,*

$$C=\langle \left(f_{0}\left(x\right)+vf_{1}\left(x\right);0\right),\left(h\left(x\right);g_{0}\left(x\right)+vg_{1}\left(x\right)\right)\rangle ,$$

*where the polynomials $f_{0}\left(x\right),f_{1}\left(x\right),g_{0}\left(x\right),g_{1}\left(x\right)\in F_{4^{e}}\left[x\right]$ and h(x)∈Re[x] with $f_{1}\left(x\right)|f_{0}\left(x\right)|\left(x^{n_{1}}-1\right)\;and\;g_{1}\left(x\right)|g_{0}\left(x\right)|\left(x^{n_{2}}-1\right).$ Moreover, $\deg\left(h\left(x\right)\right)<\deg\left(f_{0}\left(x\right)+vf_{1}\left(x\right)\right)$.*

**Lemma** **1.**  *If $C=\langle \left(f_{0}\left(x\right)+vf_{1}\left(x\right);0\right),\left(h\left(x\right);g_{0}\left(x\right)+vg_{1}\left(x\right)\right)\rangle$ is a double cyclic code of length $\left(n_{1},n_{2}\right)$ over Re, where the criteria in Theorem 1 are satisfied by the polynomials $f_{0}\left(x\right)$, $f_{1}\left(x\right)$, h(x), $g_{0}\left(x\right)$, and $g_{1}\left(x\right)$, then*

$$\left(f_{0}\left(x\right)+vf_{1}\left(x\right)\right)|\left(\frac{x^{n_{2}}-1}{g_{1}\left(x\right)}\right)h\left(x\right)\;\left(\mathrm{mod}\;x^{n_{1}}-1\right)$$

*and*

$$\left(f_{0}\left(x\right)+vf_{1}\left(x\right)\right)|\left(v\frac{x^{n_{2}}-1}{g_{0}\left(x\right)}\right)h\left(x\right)\;\left(\mathrm{mod}\;x^{n_{1}}-1\right).$$

**Corollary** **1.**  *Let $C=\langle \left(f_{0}\left(x\right)+vf_{1}\left(x\right);0\right),\left(h\left(x\right);g_{0}\left(x\right)+vg_{1}\left(x\right)\right)\rangle$ be a double cyclic code of length $\left(n_{1},n_{2}\right)$ over Re, where the criteria in Theorem 1 are satisfied by the polynomials $f_{0}\left(x\right)$, $f_{1}\left(x\right)$, h(x), $g_{0}\left(x\right)$, and $g_{1}\left(x\right)$. If $f_{0}\left(x\right)+vf_{1}\left(x\right)$ and $\frac{x^{n_{2}}-1}{g_{1}\left(x\right)}$ are co-prime over Re[x], then h(x)=0.*

**Proof.**  In view of Lemma 1, we have

$$\left(f_{0}\left(x\right)+vf_{1}\left(x\right)\right)|\left(\frac{x^{n_{2}}-1}{g_{1}\left(x\right)}\right)h\left(x\right)\;\left(\mathrm{mod}\;x^{n_{1}}-1\right).$$

Therefore,

$$(f_{0}\left(x\right)+vf_{1}\left(x\right))|h\left(x\right).$$

Since $\deg\left(h\left(x\right)\right)<\deg\left(f_{0}\left(x\right)+vf_{1}\left(x\right)\right)$, we conclude that h(x)=0. □

Some findings on separable codes over the ring Re are defined by the following propositions:

**Proposition** **2.**  *Let $C=\langle \left(f_{0}\left(x\right)+vf_{1}\left(x\right);0\right),\left(h\left(x\right);g_{0}\left(x\right)+vg_{1}\left(x\right)\right)\rangle$ be a double cyclic code of length $\left(n_{1},n_{2}\right)$ over Re, where the polynomials $f_{0}\left(x\right)$, $f_{1}\left(x\right)$, h(x), $g_{0}\left(x\right)$, $g_{1}\left(x\right)$ satisfy the conditions in Theorem 1. Then, C is a separable code iff h(x)=0.*

**Proof.**  Let h(x)=0. Then, the code *C* takes the form

$$C=\langle \left(f_{0}\left(x\right)+vf_{1}\left(x\right);0\right),\left(0;g_{0}\left(x\right)+vg_{1}\left(x\right)\right)\rangle .$$

Consequently, we have $\zeta_{n_{1}}\left(C\right)=\langle f_{0}\left(x\right)+vf_{1}\left(x\right)\rangle$ and $\zeta_{n_{2}}\left(C\right)=\langle g_{0}\left(x\right)+vg_{1}\left(x\right)\rangle$. It follows that

$$C=C_{n_{1}}\times C_{n_{2}},$$

where $C_{n_{1}}$ is a cyclic code of length $n_{1}$ over Re, generated by the ideal $\langle f_{0}\left(x\right)+vf_{1}\left(x\right)\rangle$, and the code $C_{n_{2}}$ is a cyclic code of length $n_{2}$ over Re, generated by the ideal $\langle g_{0}\left(x\right)+vg_{1}\left(x\right)\rangle$. Therefore, *C* is a separable code.

Conversely, suppose that *C* is a separable code. Now, let us define $C^{\prime }=\langle \left(f_{0}\left(x\right)+vf_{1}\left(x\right);0\right),\left(0;g_{0}\left(x\right)+vg_{1}\left(x\right)\right)\rangle$. Then, there exist codes Cn1⊆Rn1e and Cn2⊆Rn2e such that

$$C=C_{n_{1}}\times C_{n_{2}}.$$

Hence, the code *C* can be viewed as $\zeta_{n_{1}}\left(C\right)\times \zeta_{n_{2}}\left(C\right)$, where $\zeta_{n_{1}}\left(C\right)$ and $\zeta_{n_{2}}\left(C\right)$ denote the images of $C_{n_{1}}$ and $C_{n_{2}}$, respectively. Since $h\left(x\right)\in \zeta_{n_{1}}\left(C\right)$ and $0\in \zeta_{n_{2}}\left(C\right)$, it follows that

$$C^{\prime }\subseteq C.$$

Therefore,

$$\left(h\left(x\right);0\right)=k_{1}\left(x\right)*\left(f_{0}\left(x\right)+vf_{1}\left(x\right);0\right)+k_{2}\left(x\right)*\left(h\left(x\right);g_{0}\left(x\right)+vg_{1}\left(x\right)\right),$$

for some k1(x),k2(x)∈Re[x]. This implies

(1) $$\begin{array}{cc} h(x) & ={\left[k_{1}\left(x\right)\left(f_{0}\left(x\right)+vf_{1}\left(x\right)\right)+k_{2}\left(x\right)h\left(x\right)\right]}_{\left(x^{n_{1}}-1\right)},\;\mathrm{and} \end{array}$$

(2) $$\begin{array}{cc} 0 & ={\left[k_{2}\left(x\right)\left(g_{0}\left(x\right)+vg_{1}\left(x\right)\right)\right]}_{\left(x^{n_{2}}-1\right)}. \end{array}$$

From Equation (2), we have $0\equiv k_{2}\left(x\right)\left(g_{0}\left(x\right)+vg_{1}\left(x\right)\right)\;\left(\mathrm{mod}\;x^{n_{2}}-1\right)$. This implies the following possibilities for the polynomial $k_{2}\left(x\right)$:

$$k_{2}\left(x\right)=0\;\mathrm{or}\;k_{2}\left(x\right)=\frac{x^{n_{2}}-1}{g_{1}\left(x\right)}c_{1}\left(x\right)\;\mathrm{or}\;k_{2}\left(x\right)=v\frac{x^{n_{2}}-1}{g_{0}\left(x\right)}c_{2}\left(x\right),$$

for some c1(x),c2(x)∈Re[x].

**Case** **1.** If $k_{2}\left(x\right)=0$, then by Equation (1), we have

$$h\left(x\right)={\left[k_{1}\left(x\right)\left(f_{0}\left(x\right)+vf_{1}\left(x\right)\right)\right]}_{\left(x^{n_{1}}-1\right)}.$$

Hence,

$$\left(h\left(x\right);g_{0}\left(x\right)+vg_{1}\left(x\right)\right)=k_{1}\left(x\right)*\left(f_{0}\left(x\right)+vf_{1}\left(x\right);0\right)+\left(0;g_{0}\left(x\right)+vg_{1}\left(x\right)\right)\in C^{\prime }.$$

Thus, we obtain $C\subseteq C^{\prime }$.

**Case** **2.** If $k_{2}\left(x\right)=\frac{x^{n_{2}}-1}{g_{1}\left(x\right)}c_{1}\left(x\right)$, then Equation (1) gives

$$h\left(x\right)={\left[k_{1}\left(x\right)\left(f_{0}\left(x\right)+vf_{1}\left(x\right)\right)+\frac{x^{n_{2}}-1}{g_{1}\left(x\right)}c_{1}\left(x\right)h\left(x\right)\right]}_{\left(x^{n_{1}}-1\right)}.$$

It follows from Lemma 1 that there exists a polynomial j1(x)∈Re[x] such that $\frac{x^{n_{2}}-1}{g_{1}\left(x\right)}c_{1}\left(x\right)h\left(x\right)\equiv j_{1}\left(x\right)\left(f_{0}\left(x\right)+vf_{1}\left(x\right)\right)\;\left(\mathrm{mod}\;x^{n_{1}}-1\right)$. Therefore,

$$h\left(x\right)={\left[\left(k_{1}\left(x\right)+j_{1}\left(x\right)\right)\left(f_{0}\left(x\right)+vf_{1}\left(x\right)\right)\right]}_{\left(x^{n_{1}}-1\right)},$$

and so

$$\left(h\left(x\right);g_{0}\left(x\right)+vg_{1}\left(x\right)\right)=\left(k_{1}\left(x\right)+j_{1}\left(x\right)\right)*\left(f_{0}\left(x\right)+vf_{1}\left(x\right);0\right)+\left(0;g_{0}\left(x\right)+vg_{1}\left(x\right)\right)\in C^{\prime }.$$

implying again that $C\subseteq C^{\prime }$.

**Case** **3.** If $k_{2}\left(x\right)=v\frac{x^{n_{2}}-1}{g_{0}\left(x\right)}c_{2}\left(x\right)$, then from Equation (1),

$$h\left(x\right)={\left[k_{1}\left(x\right)\left(f_{0}\left(x\right)+vf_{1}\left(x\right)\right)+\left(v\frac{x^{n_{2}}-1}{g_{0}\left(x\right)}\right)c_{2}\left(x\right)h\left(x\right)\right]}_{\left(x^{n_{1}}-1\right)}.$$

By Lemma 1,

$$\left(v\frac{x^{n_{2}}-1}{g_{0}\left(x\right)}\right)c_{2}\left(x\right)h\left(x\right)\equiv j_{2}\left(x\right)\left(f_{0}\left(x\right)+vf_{1}\left(x\right)\right)\;\left(\mathrm{mod}\;x^{n_{1}}-1\right),$$

for some polynomial j2(x)∈Re[x]. Hence,

$$h\left(x\right)=\left[\left(k_{1}\left(x\right)+j_{2}\left(x\right)\right)\left(f_{0}\left(x\right)+vf_{1}\left(x\right)\right)\right]\;\left(\mathrm{mod}\;x^{n_{1}}-1\right),$$

which yields

$$\left(h\left(x\right);g_{0}\left(x\right)+vg_{1}\left(x\right)\right)=\left(k_{1}\left(x\right)+j_{2}\left(x\right)\right)*\left(f_{0}\left(x\right)+vf_{1}\left(x\right);0\right)+\left(0;g_{0}\left(x\right)+vg_{1}\left(x\right)\right)\in C^{\prime },$$

and thus $C\subseteq C^{\prime }$. In all cases, we conclude that $C=C^{\prime }=\langle \left(f_{0}\left(x\right)+vf_{1}\left(x\right);0\right),\left(0;g_{0}\left(x\right)+vg_{1}\left(x\right)\right)\rangle ,$ and therefore h(x)=0. □

The following proposition is an immediate consequence of Proposition 2.

**Proposition** **3.**  *Let $C=\langle \left(f_{0}\left(x\right)+vf_{1}\left(x\right);0\right),\left(h\left(x\right);g_{0}\left(x\right)+vg_{1}\left(x\right)\right)\rangle$ be a double cyclic code of length $\left(n_{1},n_{2}\right)$ over Re, where the criteria in Theorem 1 are satisfied by the polynomials $f_{0}\left(x\right)$, $f_{1}\left(x\right)$, h(x), $g_{0}\left(x\right)$, and $g_{1}\left(x\right)$. If $f_{0}\left(x\right)+vf_{1}\left(x\right)$ and $\frac{x^{n_{2}}-1}{g_{1}\left(x\right)}$ are co-prime in Re[x], then C is a separable code.*

## 3. Construction of Double Cyclic Codes over Re for DNA Computations

The previous section presented the construction of the generator polynomials for double cyclic codes Re. The main focus of this section is devoted to the construction of double cyclic codes specifically designed to meet the requirements of DNA computations. The following notations are used to construct cyclic DNA codes of length *n* over Re, in accordance with the framework proposed by Yildiz and Siap [16]. Let a=(a0,a1,…,an−1)∈Rne be a codeword of *n* over Re. The reverse of a is defined as $a^{r}=\left(a_{n-1},a_{n-2},\ldots ,a_{0}\right).$ The complement of a is defined as $\bar{a}=\left(\bar{a_{0}},\bar{a_{1}},\ldots ,\bar{a_{n-1}}\right),$ where $\bar{a_{i}}$ denotes the complement of $a_{i}$ in the DNA code. The reverse-complement of a is defined as $a^{rc}=\left(\bar{a_{n-1}},\bar{a_{n-2}},\ldots ,\bar{a_{0}}\right).$

A code *C* of length *n* over Re is called reversible if for any a∈C, $a^{r}\in C$, complement if for any a∈C, $\bar{a}\in C$, reversible-complement if for any a∈C, $a^{rc}\in C$. Additionally, a cyclic code *C* is referred to as a cyclic DNA code when, for every a∈C with $a\neq a^{rc}$ and $a^{rc}$ also belongs to *C*. The reciprocal polynomial of f(x)=f0+f1x+…+ftxt∈Re[x], with $f_{t}\neq 0$ is defined as follows:

$$f^{*}\left(x\right)=x^{t}f\left(x^{-1}\right)=f_{t}+f_{t-1}x+\ldots +f_{0}x^{t}.$$

It is evident that if $f_{0}\neq 0$, then $\deg\left(f^{*}\left(x\right)\right)=\deg\left(f\left(x\right)\right)$, else $\deg\left(f^{*}\left(x\right)\right)\leq \deg\left(f\left(x\right)\right)$. Moreover, the polynomial f(x) is said to be a self reciprocal if $f^{*}\left(x\right)=f\left(x\right)$.

By the same argument as in Abualrub et al. [11] Lemma 4, we obtain the following lemma.

**Lemma** **2.**  *Let f(x) and g(x) be two polynomials in Re[x] such that deg(f(x))≥deg(g(x)) with i=deg(f(x))−deg(g(x)). Then,*

*(i)*  ${\left[f\left(x\right)+g\left(x\right)\right]}^{*}=f^{*}\left(x\right)+x^{i}g^{*}\left(x\right)$,
*(ii)*  ${\left[f\left(x\right)g\left(x\right)\right]}^{*}=f^{*}\left(x\right)g^{*}\left(x\right)$.

For any odd length *n*, Guenda and Gulliver [13] investigated into cyclic DNA codes over the ring $R=F_{2}+vF_{2}\;\mathrm{with}\;v^{2}=0$. They introduced a bijective mapping η between the elements of R and the DNA nucleotides *A*, *T*, *G*, *C* as follows:

0⟼A,1⟼G,v⟼T,1+v⟼C.

For our study, the present section will consistently use the same bijective map η. Based on the linkage properties of DNA bases, the following equalities are observed: $\bar{0}=v,\;\bar{1}=1+v,\;\bar{v}=0,\;\mathrm{and}\;\bar{1+v}=1.$ For any a,b∈Re, we obtain $a+\bar{a}=v,\;\bar{a+b}=\bar{a}+\bar{b}+v.$ Furthermore, for any $a\in F_{4^{e}}$, we have $v+\bar{va}=va.$

The next theorem concerning reversible and reversible-complement codes is an analogous result as presented in the work of Guenda and Gulliver [13].

**Theorem** **2.**  *Let $C=\langle f_{0}\left(x\right),vf_{1}\left(x\right)\rangle =\langle f_{0}\left(x\right)+vf_{1}\left(x\right)\rangle$ be a cyclic code of odd length n over Re, where $f_{0}\left(x\right),f_{1}\left(x\right)\in F_{4^{e}}\left[x\right]$ with $f_{1}\left(x\right)|f_{0}\left(x\right)|\left(x^{n}-1\right)$. Then,*

*(i)*  *the code C is reversible if the polynomials $f_{0}\left(x\right)$ and $f_{1}\left(x\right)$ are self-reciprocal.*
*(ii)*  *the code C is reversible-complement if the polynomials $f_{0}\left(x\right)$ and $f_{1}\left(x\right)$ are self-reciprocal and C contains the codeword $v+vx+\ldots +vx^{n-1}$.*

**Remark** **1**  ([31])**.** *Let u=(a;b)=(a0,a1,…,an1−1;b0,b1,…,bn2−1)∈Rn1,n2e. Then,*

*(i)*  *the reverse of u is defined as $u^{r}=\left(a_{n_{1}-1},a_{n_{1}-2},\ldots ,a_{0};b_{n_{2}-1},b_{n_{2}-2},\ldots ,b_{0}\right)$, that is, $u^{r}=\left(a^{r};b^{r}\right)$,*
*(ii)*  *the complement of u is defined as $\bar{u}=\left(\bar{a_{0}},\bar{a_{1}},\ldots ,\bar{a_{n_{1}-1}};\bar{b_{0}},\bar{b_{1}},\ldots ,\bar{b_{n_{2}-1}}\right)$, that is, $\bar{u}=\left(\bar{a};\bar{b}\right)$,*
*(iii)*  *the reverse-complement of u is defined as $u^{rc}=\left(a_{n_{1}-1},a_{n_{1}-2},\ldots ,a_{0};b_{n_{2}-1},b_{n_{2}-2},\ldots ,b_{0}\right),$ that is, $u^{rc}=\left(a^{rc};b^{rc}\right).$*

In the continuation of earlier studies on DNA codes formulated within cyclic codes over the ring R (see [10,13]) and on cyclic codes defined over mixed alphabets [19], we extend the framework by introducing reversible, complement, and reversible-complement structures for double cyclic codes of length $\left(n_{1},n_{2}\right)$ over the ring Re, which prove to be highly applicable in DNA-based computations.

**Definition** **2.**  *Let C be a double cyclic code of length $\left(n_{1},n_{2}\right)$ over the ring Re. Then, C is said to be reversible if for any u∈C, $u^{r}\in C$, complement if for any u∈C, $\bar{u}\in C$, reversible-complement if for any u∈C, $u^{rc}\in C$.*

The following is a definition of the double cyclic DNA codes of length $\left(n_{1},n_{2}\right)$ over the ring Re.

**Definition** **3.**  *Let C be a linear code of length $n=n_{1}+n_{2}$ defined over the ring Re. We call C a double cyclic DNA code of length $\left(n_{1},n_{2}\right)$ over Re if the code C forms a double cyclic code of the same length over Re, and for each u∈C, the conditions $u\neq u^{rc}$ and $u^{rc}\in C$ are satisfied.*

We break down the investigation into three subsections to make it easier to analyze double cyclic DNA codes of length $\left(n_{1},n_{2}\right)$ over Re:

(i) The initial subsection concentrates on the investigation of the reversibility of double cyclic codes of length $\left(n_{1},n_{2}\right)$ over Re.
(ii) The second subsection examines the reversible-complement property and illustrate several examples of DNA codes derived through double cyclic DNA codes of length $\left(n_{1},n_{2}\right)$ defined over Re.
(iii) In the third subsection, the appropriate Gray map for Re=F4e+vF4e, where $v^{2}=0$, is the subject of discussion.

### 3.1. Reversible Codes over Re

The analysis in this subsection focuses on characterizing the reversibility of double cyclic codes through necessary and sufficient conditions. In view of Remark 1 mentioned above, the following result is straightforward.

**Proposition** **4.**  *Let u,w∈Rn1,n2e, and k∈Re. Then,*

*(i)* 
${\left[u+w\right]}^{r}=u^{r}+w^{r},$

*(ii)* 
${\left[k*u\right]}^{r}=k*u^{r}.$

**Remark** **2.**  *For any u=(a0,a1,…,an1−1;b0,b1,…,bn2−1)∈Rn1,n2e, the vector u is associated with the polynomial u(x)=(a(x);b(x))=(a0+a1x+…+an1−1xn1−1;b0+b1x+…+bn2−1xn2−1)∈Rn1,n2e. Since by Remark 1, $u^{r}=\left(a_{n_{1}-1},a_{n_{1}-2},\ldots ,a_{0};b_{n_{2}-1},b_{n_{2}-2},\ldots ,b_{0}\right)$, we obtain*

$$\begin{array}{ccc} u^{r}\left(x\right) & = & \left(a_{n_{1}-1}+a_{n_{1}-2}x+\ldots +a_{0}x^{n_{1}-1};b_{n_{2}-1}+b_{n_{2}-2}x+\ldots +b_{0}x^{n_{2}-1}\right)\\ & = & \left(x^{n_{1}-1-\deg\left(a\left(x\right)\right)}a^{*}\left(x\right);x^{n_{2}-1-\deg\left(b\left(x\right)\right)}b^{*}\left(x\right)\right), \end{array}$$

where $a^{*}\left(x\right)$ and $b^{*}\left(x\right)$ are the reciprocal polynomials of a(x) and b(x), respectively. This shows that the reverse of u(x) corresponds to the polynomial

$$u^{r}\left(x\right)=\left(x^{n_{1}-1-\deg\left(a\left(x\right)\right)}a^{*}\left(x\right);x^{n_{2}-1-\deg\left(b\left(x\right)\right)}b^{*}\left(x\right)\right).$$

*Additionally, the reverse of u(x) can be written as ${\left[u\left(x\right)\right]}^{r}=u^{r}\left(x\right)$.*

Let *C* be a double cyclic code of length $\left(n_{1},n_{2}\right)$ over the ring Re. Define the projection maps

σn1:C→Rn1ebyσn1(a;b)=afor all (a;b)∈C,

and

σn2:C→Rn2ebyσn2(a;b)=bfor all (a;b)∈C.

The codes $\sigma_{n_{1}}\left(C\right)$ and $\sigma_{n_{2}}\left(C\right)$ are both reversible cyclic codes over Re with lengths $n_{1}$ and $n_{2}$ provided that *C* is a reversible code. Moreover, if *C* is separable, then it can be expressed as $C=C_{n_{1}}\times C_{n_{2}}$, with Cn1⊆Rn1e and Cn2⊆Rn2e. Hence, $\sigma_{n_{1}}\left(C\right)=C_{n_{1}}$ and $\sigma_{n_{2}}\left(C\right)=C_{n_{2}}$. It follows that for a separable code *C*, reversibility holds precisely when both $C_{n_{1}}$ and $C_{n_{2}}$ are reversible cyclic codes of lengths $n_{1}$ and $n_{2}$ over Re and conversely. Consequently, this leads us to the following proposition:

**Proposition** **5.**  *Let C be a double cyclic code of length $\left(n_{1},n_{2}\right)$ over Re and assume that $C=\langle \left(f_{0}\left(x\right)+vf_{1}\left(x\right),0\right);\left(0,g_{0}\left(x\right)+vg_{1}\left(x\right)\right)\rangle$ is a separable code, where the criteria in Theorem 1 are satisfied by the polynomials $f_{0}\left(x\right),f_{1}\left(x\right),g_{0}\left(x\right),$ and $g_{1}\left(x\right)$. Then, the code C is reversible iff the polynomials $f_{0}\left(x\right),f_{1}\left(x\right),g_{0}\left(x\right),$ and $g_{1}\left(x\right)$ are self-reciprocal.*

**Proof.**  Let the polynomials $f_{0}\left(x\right),f_{1}\left(x\right),g_{0}\left(x\right),$ and $g_{1}\left(x\right)$ be self-reciprocal and given that *C* is a separable code. Then, it follows $C=C_{n_{1}}\times C_{n_{2}}$, with $C_{n_{1}}$ is a cyclic code of length $n_{1}$ over Re generated by $f_{0}\left(x\right)+vf_{1}\left(x\right)$, and $C_{n_{2}}$ is a cyclic code of length $n_{2}$ over Re generated by $g_{0}\left(x\right)+vg_{1}\left(x\right)$. According to Theorem 2, the component codes $C_{n_{1}}$ and $C_{n_{2}}$ are reversible cyclic codes of lengths $n_{1}$ and $n_{2}$, respectively, over the ring Re. For any $v=\left(a;b\right)\in C=C_{n_{1}}\times C_{n_{2}}$, we have $a\in C_{n_{1}}$ and $b\in C_{n_{2}}$. As $C_{n_{1}}$ and $C_{n_{2}}$ are reversible, it follows that $a^{r}\in C_{n_{1}}$ and $b^{r}\in C_{n_{2}}$. Thus,

$$u^{r}=\left(a^{r};b^{r}\right)\in C_{n_{1}}\times C_{n_{2}}=C.$$

Hence, *C* is a reversible code.

Conversely, assume that the code *C* is reversible. In this case, $\sigma_{n_{1}}\left(C\right)$ and $\sigma_{n_{2}}\left(C\right)$ are reversible cyclic codes of length $n_{1}$ and $n_{2}$ over Re, respectively. Given that $\zeta_{n_{1}}\left(C\right)=\langle f_{0}\left(x\right)+vf_{1}\left(x\right)\rangle$, “it follows that $\sigma_{n_{1}}\left(C\right)$ is a cyclic code of length $n_{1}$ over Re with generator $f_{0}\left(x\right)+vf_{1}\left(x\right)$. By Theorem 2, the polynomials $f_{0}\left(x\right)$ and $f_{1}\left(x\right)$ are self reciprocal polynomials. In a similar way, $\sigma_{n_{2}}\left(C\right)$ is a cyclic code of length *b* over Re with generator $g_{0}\left(x\right)+vg_{1}\left(x\right)$. In view of Theorem 2, we conclude that $g_{0}\left(x\right)$ and $g_{1}\left(x\right)$ are self-reciprocal. □

**Proposition** **6.**  *Let u(x), w(x), and (a(x);b(x)) be elements in Rn1,n2e and $k\in Z^{+}$. Then,*

*(i)*  *${\left[u\left(x\right)+w\left(x\right)\right]}^{r}=u^{r}\left(x\right)+w^{r}\left(x\right)$,*
*(ii)* 
${\left(x^{k}a\left(x\right);x^{k}b\left(x\right)\right)}^{r}=x^{\left(p+1\right)n_{1}-1-\deg\left(x^{k}a\left(x\right)\right)}*\left(a^{*}\left(x\right);0\right)+x^{\left(q+1\right)n_{2}-1-\deg\left(x^{k}b\left(x\right)\right)}$ $*\;(0;b^{*}\left(x\right)),$

*where p,q are either zero or the least positive integers such that*

$$pn_{1}-\deg\left(x^{k}a\left(x\right)\right)+\deg\left({\left[x^{k}a\left(x\right)\right]}_{\left(x^{n_{1}}-1\right)}\right)\geq 0$$

*and*

$$qn_{2}-\deg\left(x^{k}b\left(x\right)\right)+\deg\left({\left[x^{k}b\left(x\right)\right]}_{\left(x^{n_{2}}-1\right)}\right)\geq 0.$$

**Proof.**  We establish statement (i) directly. Now, we proceed to prove statement (ii). Consider an element (a(x);b(x))∈Rn1,n2e, where $k\in Z^{+}$. The proof is carried out by considering the following four cases:

**Case** **1.** If $\deg\left(x^{k}a\left(x\right)\right)\geq n_{1}$ and $\deg\left(x^{k}b\left(x\right)\right)\geq n_{2}$, then we have

$$x^{k}a\left(x\right)=\left(x^{n_{1}}-1\right)q_{1}\left(x\right)+l_{1}\left(x\right)\;\mathrm{and}\;x^{k}b\left(x\right)=\left(x^{n_{2}}-1\right)q_{2}\left(x\right)+l_{2}\left(x\right),$$

where q1(x),q2(x),l1(x),l2(x)∈Re[x], $\deg\left(l_{1}\left(x\right)\right)\leq n_{1}-1$, and $\deg\left(l_{2}\left(x\right)\right)\leq n_{2}-1$. Consequently, $a^{*}\left(x\right)={\left(x^{k}a\left(x\right)\right)}^{*}=\left(x^{n_{1}}-1\right)q_{1}^{*}\left(x\right)+x^{i}l_{1}^{*}\left(x\right)$ and $b^{*}\left(x\right)={\left(x^{k}b\left(x\right)\right)}^{*}=\left(x^{n_{2}}-1\right)q_{2}^{*}\left(x\right)+x^{j}l_{2}^{*}\left(x\right),$ where $i=\deg\left(\left(x^{n_{1}}-1\right)q_{1}\left(x\right)\right)-\deg\left(l_{1}\left(x\right)\right)$ and $j=\deg\left(\left(x^{n_{2}}-1\right)q_{2}\left(x\right)\right)-\deg\left(l_{2}\left(x\right)\right)$. Let $p,q\in Z^{+}$ be the least positive integers such that $qn_{1}-i\geq 0$ and $qn_{2}-j\geq 0$. Then, it follows that

$$l_{1}^{*}\left(x\right)={\left[x^{pn_{1}-i}a^{*}\left(x\right)\right]}_{\left(x^{n_{1}}-1\right)}\;\mathrm{and}\;l_{2}^{*}\left(x\right)={\left[x^{qn_{2}-j}b^{*}\left(x\right)\right]}_{\left(x^{n_{2}}-1\right)}.$$

Now consider

$${\left(x^{k}a\left(x\right);x^{k}b\left(x\right)\right)}^{r}={\left(l_{1}\left(x\right);l_{2}\left(x\right)\right)}^{r}=\left(x^{n_{1}-1-\deg\left(l_{1}\left(x\right)\right)}l_{1}^{*}\left(x\right);x^{n_{2}-1-\deg\left(l_{2}\left(x\right)\right)}l_{2}^{*}\left(x\right)\right).$$

Substituting the expressions of $l_{1}^{*}\left(x\right)$ and $l_{2}^{*}\left(x\right)$ in the relation ${\left(x^{k}a\left(x\right);x^{k}b\left(x\right)\right)}^{r}$, we obtain

$$\begin{array}{cc} \; & {\left(x^{k}a\left(x\right);x^{k}b\left(x\right)\right)}^{r}\\ \; & \;=\left(x^{n_{1}-1-\deg\left(l_{1}\left(x\right)\right)}x^{pn_{1}-i}a^{*}\left(x\right);0\right)+\left(0;x^{n_{2}-1-\deg\left(l_{2}\left(x\right)\right)}x^{qn_{2}-j}b^{*}\left(x\right)\right)\\ \; & \;=x^{\left(p+1\right)n_{1}-1-\deg\left(\left(x^{n_{1}}-1\right)q_{1}\left(x\right)\right)}*\left(a^{*}\left(x\right);0\right)+x^{\left(q+1\right)n_{2}-1-\deg\left(\left(x^{n_{2}}-1\right)q_{2}\left(x\right)\right)}*\left(0;b^{*}\left(x\right)\right). \end{array}$$

Since $\deg\left(\left(x^{n_{1}}-1\right)q_{1}\left(x\right)\right)=\deg\left(x^{k}a\left(x\right)\right)$ and $\deg\left(\left(x^{n_{2}}-1\right)q_{2}\left(x\right)\right)=\deg\left(x^{k}b\left(x\right)\right)$, we arrive at ${\left(x^{k}a\left(x\right);x^{k}b\left(x\right)\right)}^{r}=x^{\left(p+1\right)n_{1}-1-\deg\left(x^{k}a\left(x\right)\right)}*\left(a^{*}\left(x\right);0\right)+x^{\left(q+1\right)n_{2}-1-\deg\left(x^{k}b\left(x\right)\right)}*\left(0;b^{*}\left(x\right)\right).$

**Case** **2.** If $\deg\left(x^{k}a\left(x\right)\right)\leq n_{1}-1$ and $\deg\left(x^{k}b\left(x\right)\right)\geq n_{2}$, then

$$x^{k}b\left(x\right)=\left(x^{n_{2}}-1\right)q_{2}\left(x\right)+l_{2}\left(x\right),$$

where q2(x),l2(x)∈Re[x] and $\deg\left(l_{2}\left(x\right)\right)\leq n_{2}-1$. Consequently,

$$a^{*}\left(x\right)={\left(x^{k}a\left(x\right)\right)}^{*}\;\mathrm{and}\;b^{*}\left(x\right)=\left(x^{n_{2}}-1\right)q_{2}^{*}\left(x\right)+x^{j}l_{2}^{*}\left(x\right),$$

where $j=\deg\left(\left(x^{n_{2}}-1\right)q_{2}\left(x\right)\right)-\deg\left(l_{2}\left(x\right)\right)$. Let $q\in Z^{+}$ be the least positive integer such that $qn_{2}-j\geq 0$. Then, we obtain

$$l_{2}^{*}\left(x\right)={\left[x^{qn_{2}-j}b^{*}\left(x\right)\right]}_{\left(x^{n_{2}}-1\right)}.$$

Now, we compute

$$\begin{array}{cc} {\left(x^{k}a\left(x\right);x^{k}b\left(x\right)\right)}^{r} & ={\left(x^{k}a\left(x\right);l_{2}\left(x\right)\right)}^{r}\\ \; & =(x^{n_{1}-1-\deg\left(x^{k}a\left(x\right)\right)}{\left(x^{k}a\left(x\right)\right)}^{*};x^{n_{2}-1-\deg\left(l_{2}\left(x\right)\right)}l_{2}^{*}\left(x\right))\\ \; & =\left(x^{n_{1}-1-\deg\left(x^{k}a\left(x\right)\right)}a^{*}\left(x\right);0\right)+\left(0;x^{n_{2}-1-\deg\left(l_{2}\left(x\right)\right)}x^{qn_{2}-j}b^{*}\left(x\right)\right)\\ \; & =x^{n_{1}-1-\deg\left(x^{k}a\left(x\right)\right)}*\left(a^{*}\left(x\right);0\right)+x^{\left(q+1\right)n_{2}-1-\deg\left(\left(x^{n_{2}}-1\right)q_{2}\left(x\right)\right)}*\left(0;b^{*}\left(x\right)\right). \end{array}$$

Since $\deg\left(\left(x^{n_{2}}-1\right)q_{2}\left(x\right)\right)=\deg\left(x^{k}b\left(x\right)\right)$, so we obtain

$${\left(x^{k}a\left(x\right);x^{k}b\left(x\right)\right)}^{r}=x^{n_{1}-1-\deg\left(x^{k}a\left(x\right)\right)}*\left(a^{*}\left(x\right);0\right)+x^{\left(q+1\right)n_{2}-1-\deg\left(x^{k}b\left(x\right)\right)}*\left(0;b^{*}\left(x\right)\right).$$

**Case** **3.** When $\deg\left(x^{k}a\left(x\right)\right)\leq n_{1}-1$ and $\deg\left(x^{k}b\left(x\right)\right)\leq n_{2}-1$, then we have

$${\left(x^{k}a\left(x\right)\right)}^{*}=a^{*}\left(x\right)\;\mathrm{and}\;{\left(x^{k}b\left(x\right)\right)}^{*}=b^{*}\left(x\right),\;\mathrm{Thus},$$

$$\begin{array}{cc} {\left(x^{k}a\left(x\right);x^{k}b\left(x\right)\right)}^{r} & =(x^{n_{1}-1-\deg\left(x^{k}a\left(x\right)\right)}\cdot {\left(x^{k}a\left(x\right)\right)}^{*};x^{n_{2}-1-\deg\left(x^{k}b\left(x\right)\right)}\cdot {\left(x^{k}b\left(x\right)\right)}^{*})\\ \; & =(x^{n_{1}-1-\deg\left(x^{k}a\left(x\right)\right)}\cdot a^{*}\left(x\right);x^{n_{2}-1-\deg\left(x^{k}b\left(x\right)\right)}\cdot b^{*}\left(x\right))\\ \; & =x^{n_{1}-1-\deg\left(x^{k}a\left(x\right)\right)}*\left(a^{*}\left(x\right);0\right)+x^{n_{2}-1-\deg\left(x^{k}b\left(x\right)\right)}*\left(0;b^{*}\left(x\right)\right). \end{array}$$

This implies that

$${\left(x^{k}a\left(x\right);x^{k}b\left(x\right)\right)}^{r}=x^{\left(p+1\right)n_{1}-1-\deg\left(x^{k}a\left(x\right)\right)}*\left(a^{*}\left(x\right);0\right)+x^{\left(q+1\right)n_{2}-1-\deg\left(x^{k}b\left(x\right)\right)}*\left(0;b^{*}\left(x\right)\right),$$

where p=q=0.

**Case** **4.** When $\deg\left(x^{k}a\left(x\right)\right)\geq n_{1}$ and $\deg\left(x^{k}b\left(x\right)\right)\leq n_{2}-1$, then the proof proceeds analogously to Case 2. Thus, considering all four cases, we conclude that

$${\left(x^{k}a\left(x\right);x^{k}b\left(x\right)\right)}^{r}=x^{\left(p+1\right)n_{1}-1-\deg\left(x^{k}a\left(x\right)\right)}*\left(a^{*}\left(x\right);0\right)+x^{\left(q+1\right)n_{2}-1-\deg\left(x^{k}b\left(x\right)\right)}*\left(0;b^{*}\left(x\right)\right),$$

where p,q are either 0 or the least positive integers satisfying $pn_{1}-\deg\left(x^{k}a\left(x\right)\right)+\deg\left({\left[x^{k}a\left(x\right)\right]}_{\left(x^{n_{1}}-1\right)}\right)\geq 0,\;\mathrm{and}\;qn_{2}-\deg\left(x^{k}b\left(x\right)\right)+\deg\left({\left[x^{k}b\left(x\right)\right]}_{\left(x^{n_{2}}-1\right)}\right)\geq 0.$ □

**Corollary** **2.**  Let (a(x);b(x))∈Rn1,n2e with $k\in Z^{+}$. Suppose there exists a non-negative integer $n_{3}$ such that $n_{2}=\left(2n_{3}+1\right)n_{1}$ with $\deg\left(b\left(x\right)\right)=2n_{3}n_{1}+\deg\left(a\left(x\right)\right)$. Then, ${\left(x^{k}a\left(x\right);x^{k}b\left(x\right)\right)}^{r}=x^{\left(\ell \left(2n_{3}+1\right)+1\right)n_{1}-1-\deg\left(x^{k}a\left(x\right)\right)}*\left(a^{*}\left(x\right);b^{*}\left(x\right)\right),$ where ℓ is either zero or the smallest positive integer satisfying

$$\ell n_{1}-\deg\left(x^{k}a\left(x\right)\right)+\deg\left({\left[x^{k}a\left(x\right)\right]}_{\left(x^{n_{1}}-1\right)}\right)\geq 0,$$

*and*

$$\ell n_{2}-\deg\left(x^{k}b\left(x\right)\right)+\deg\left({\left[x^{k}b\left(x\right)\right]}_{\left(x^{n_{2}}-1\right)}\right)\geq 0.$$

**Proof.**  The proof directly follows from the Proposition 6. □

**Example** **2.**  *For e=2, we have R2=F16+vF16, where $v^{2}=0$. Let (a(x);b(x))=(1+(1+w3)x+w2x2;w2+wx2+(1+w+w3)x3)∈R5,72. Consider ${\left(x^{2}a\left(x\right);x^{2}b\left(x\right)\right)}^{r}={\left(x^{2}\left(1+\left(1+w^{3}\right)x+w^{2}x^{2}\right);x^{2}\left(1+x^{2}+x^{3}\right)\right)}^{r}$. Since $\deg\left(x^{2}a\left(x\right)\right)=\deg\left(x^{2}+\left(1+w^{3}\right)x^{3}+w^{2}x^{4}\right)=4$, $\deg\left({\left[x^{2}a\left(x\right)\right]}_{\left(x^{5}-1\right)}\right)=\deg\left(x^{2}+\left(1+w^{3}\right)x^{3}+w^{2}x^{4}\right)=4$, $\deg\left(x^{2}b\left(x\right)\right)=\deg\left(w^{2}x^{2}+wx^{4}+\left(1+w+w^{3}\right)x^{5}\right)=5$, and $\deg\left({\left[x^{2}b\left(x\right)\right]}_{\left(x^{7}-1\right)}\right)=\deg\left(w^{2}x^{2}+wx^{4}+\left(1+w+w^{3}\right)x^{5}\right)=5$, we obtain that p=0 such that 0(5)−4+4≥0 and q=0. By using Proposition 6, we obtain that*

$$\begin{array}{cc} \; & {\left(x^{2}a\left(x\right);x^{2}b\left(x\right)\right)}^{r}\\ \; & =x^{\left(p+1\right)n_{1}-1-\deg\left(x^{2}a\left(x\right)\right)}*\left(a^{*}\left(x\right);0\right)+x^{\left(q+1\right)n_{2}-1-\deg\left(x^{2}b\left(x\right)\right)}*\left(0;b^{*}\left(x\right)\right)\\ \; & =x^{5-1-4}*\left(a^{*}\left(x\right);0\right)+x^{7-1-5}*\left(0;b^{*}\left(x\right)\right)\\ \; & =1*\left(w^{2}+\left(1+w^{3}\right)x+x^{2};0\right)+x*\left(0;\left(1+w+w^{3}\right)+wx+w^{2}x^{3}\right)\\ \; & =(w^{2}+\left(1+w^{3}\right)x+x^{2}\;mod\left(x^{5}-1\right);\left(1+w+w^{3}\right)x+wx^{2}+w^{2}x^{4}\;mod\left(x^{7}-1\right))\\ \; & =\left(w^{2}+\left(1+w^{3}\right)x+x^{2};\left(1+w+w^{3}\right)x+wx^{2}+w^{2}x^{4}\right). \end{array}$$

*On the other hand, by performing a direct computation, we obtain*

$$\begin{array}{cc} \; & {\left(x^{2}a\left(x\right);x^{2}b\left(x\right)\right)}^{r}\\ \; & ={\left(x^{2}\left(1+\left(1+w^{3}\right)x+w^{2}x^{2}\right),x^{2}\left(w^{2}+wx^{2}+\left(1+w+w^{3}\right)x^{3}\right)\right)}^{r}\\ \; & ={\left(x^{2}+\left(1+w^{3}\right)x^{3}+w^{2}x^{4}\;mod\left(x^{5}-1\right);w^{2}x^{2}+wx^{4}+\left(1+w+w^{3}\right)x^{5}\;mod\left(x^{7}-1\right)\right)}^{r}\\ \; & ={\left(x^{2}+\left(1+w^{3}\right)x^{3}+w^{2}x^{4},w^{2}x^{2}+wx^{4}+\left(1+w+w^{3}\right)x^{5}\right)}^{r}\\ \; & =\left(x^{5-1-4}{\left(x^{2}+\left(1+w^{3}\right)x^{3}+w^{2}x^{4}\right)}^{*};x^{7-1-5}{\left(w^{2}x^{2}+wx^{4}+\left(1+w+w^{3}\right)x^{5}\right)}^{*}\right)\\ \; & =\left(w^{2}+\left(1+w^{3}\right)x+x^{2};\left(1+w+w^{3}\right)x+wx^{2}+w^{2}x^{4}\right). \end{array}$$

The following example illustrates the case when both *p* and *q* are non-zero.

**Example** **3.**  *For e=1, we have R1=F4+vF4, where $v^{2}=0$. Let (a(x),b(x))=(1+wx3+x4,1+x+(1+w)x6)∈R5,71. Consider ${\left(xa\left(x\right),xb\left(x\right)\right)}^{r}={\left(x\left(1+wx^{3}+x^{4}\right),x\left(1+x+\left(1+w\right)x^{6}\right)\right)}^{r}$. Since $\deg\left(xa\left(x\right)\right)=\deg\left(x+wx^{4}+x^{5}\right)=5$, $\deg\left({\left[xa\left(x\right)\right]}_{\left(x^{5}-1\right)}\right)=\deg\left(1+x+wx^{4}\right)=4$, $\deg\left(xb\left(x\right)\right)=\deg\left(x+x^{2}+\left(1+w\right)x^{7}\right)=7$, and $\deg\left({\left[xb\left(x\right)\right]}_{\left(x^{7}-1\right)}\right)=\deg{\left[x+x^{2}+\left(1+w\right)x^{7}\right]}_{\left(x^{7}-1\right)}=\deg\left(\left(1+w\right)+x+x^{2}\right)=2$. Then, by using Proposition 6, we obtain that p=1 such that 1(5)−5+4≥0 and q=1. Moreover, we have*

$$\begin{array}{ccc} {\left(xa\left(x\right);xb\left(x\right)\right)}^{r} & = & x^{\left(p+1\right)n_{1}-1-\deg\left(xa\left(x\right)\right)}*\left(a^{*}\left(x\right);0\right)+x^{\left(q+1\right)n_{2}-1-\deg\left(xb\left(x\right)\right)}*\left(0;b^{*}\left(x\right)\right)\\ & = & x^{10-1-5}*\left(a^{*}\left(x\right);0\right)+x^{14-1-7}*\left(0;b^{*}\left(x\right)\right)\\ & = & x^{4}*\left(1+wx+x^{4};0\right)+x^{6}*\left(0;\left(1+w\right)+x^{5}+x^{6}\right)\\ & = & \left(x^{4}+wx^{5}+x^{8}\;\mathrm{mod}\;\left(x^{5}-1\right);\left(1+w\right)x^{6}+x^{11}+x^{12}\;\mathrm{mod}\;\left(x^{7}-1\right)\right)\\ & = & (w+x^{3}+x^{4};x^{4}+x^{5}+\left(1+w\right)x^{6}). \end{array}$$

*However, a direct computation yields that*

$$\begin{array}{ccc} {\left(xa\left(x\right);xb\left(x\right)\right)}^{r} & = & {\left(x\left(1+wx^{3}+x^{4}\right),x\left(1+x+\left(1+w\right)x^{6}\right)\right)}^{r}\\ & = & {\left(x+wx^{4}+x^{5}\;\mathrm{mod}\;\left(x^{5}-1\right);x+x^{2}+\left(1+w\right)x^{7}\;\mathrm{mod}\;\left(x^{7}-1\right)\right)}^{r}\\ & = & {\left(1+x+wx^{4},1+x+x^{2}\right)}^{r}\\ & = & \left(x^{5-1-4}{\left(1+x+wx^{4}\right)}^{*};x^{7-1-2}{\left(\left(1+w\right)+x+x^{2}\right)}^{*}\right)\\ & = & \left(w+x^{3}+x^{4};x^{4}\left(1+x+\left(1+w\right)x^{2}\right)\right)\\ & = & (w+x^{3}+x^{4};x^{4}+x^{5}+\left(1+w\right)x^{6}). \end{array}$$

In the following theorem, we investigate the reversal property of a non-separable double cyclic code *C* of length $\left(n_{1},n_{2}\right)$ over the ring Re.

**Theorem** **3.**  *Let $C=\langle \left(f_{0}\left(x\right)+vf_{1}\left(x\right);0\right),\left(h\left(x\right);g_{0}\left(x\right)+vg_{1}\left(x\right)\right)\rangle$ be a double cyclic code of length $\left(n_{1},n_{2}\right)$ over Re, where the criteria in Theorem 1 are satisfied by the polynomials $f_{0}\left(x\right)$, $f_{1}\left(x\right)$, $g_{0}\left(x\right)$, $g_{1}\left(x\right)$, and h(x). Then, $f_{0}\left(x\right)$, $f_{1}\left(x\right)$, $g_{0}\left(x\right)$, and $g_{1}\left(x\right)$ are self-reciprocal polynomials if the code C is reversible.*

**Proof.**  Assume that *C* is reversible. Subsequently, $\zeta_{n_{2}}\left(C\right)=\langle g_{0}\left(x\right)+vg_{1}\left(x\right)\rangle$ is a reversible cyclic code of length $n_{2}$ over Re. Based on Theorem 2, it is evident that both $g_{0}\left(x\right)$ and $g_{1}\left(x\right)$ are self-reciprocal. Our assertion is that the code $\langle \left(f_{0}\left(x\right)+vf_{1}\left(x\right);0\right)\rangle$ constitutes a reversible double cyclic code of length $\left(n_{1},n_{2}\right)$ over the ring Re. Since $\langle \left(f_{0}\left(x\right)+vf_{1}\left(x\right);0\right)\rangle$ is an Re[x]-submodule of Rn1,n2e, so it is a double cyclic code of length $n_{1}$ over Re. Let us assume, contrary to our claim, that $I=\langle \left(f_{0}\left(x\right)+vf_{1}\left(x\right);0\right)\rangle$ is not reversible. Then, there exists u(x)∈I⊆C such that $u^{r}\left(x\right)\notin I$. Since $u\left(x\right)=k\left(x\right)*(f_{0}\left(x\right)+vf_{1}\left(x\right);0)$, we have

$$u^{r}\left(x\right)=\left(x^{n_{1}-1-\deg\left(l\left(x\right)\right)}l^{*}\left(x\right);0\right),$$

where ${\left[k\left(x\right)\left(f_{0}\left(x\right)+vf_{1}\left(x\right)\right)\right]}_{\left(x^{n_{1}}-1\right)}=l\left(x\right)$. Thus, we have

$$u^{r}\left(x\right)=\left(x^{n_{1}-1-\deg\left(l\left(x\right)\right)}l^{*}\left(x\right);0\right)=k_{1}\left(x\right)*\left(f_{0}\left(x\right)+vf_{1}\left(x\right);0\right)+k_{2}\left(x\right)*\left(h\left(x\right);g_{0}\left(x\right)+vg_{1}\left(x\right)\right),$$

for some k1(x),k2(x)∈Re[x] with $k_{2}\left(x\right)\neq 0$. This demonstrates that

(3) $${\left[k_{1}\left(x\right)\left(f_{0}\left(x\right)+vf_{1}\left(x\right)\right)+k_{2}\left(x\right)h\left(x\right)\right]}_{\left(x^{n_{1}}-1\right)}=x^{n_{1}-1-\deg\left(l\left(x\right)\right)}l^{*}\left(x\right),$$

(4) $$[k_{2}\left(x\right)\left(g_{0}\left(x\right)+vg_{1}\left(x\right)\right){]}_{\left(x^{n_{2}}-1\right)}=0.$$

Since $k_{2}\left(x\right)\neq 0$, there are two cases: either $k_{2}\left(x\right)=\frac{x^{n_{2}}-1}{g_{1}\left(x\right)}l_{1}\left(x\right)$ or $k_{2}\left(x\right)=v\frac{x^{n_{2}}-1}{g_{0}\left(x\right)}l_{2}\left(x\right)$, where l1(x),l2(x)∈Re[x].

**Case** **1.** If $k_{2}\left(x\right)=\frac{x^{n_{2}}-1}{g_{1}\left(x\right)}l_{1}\left(x\right)$, then

$$k_{2}\left(x\right)h\left(x\right)=\frac{x^{n_{2}}-1}{g_{1}\left(x\right)}l_{1}\left(x\right)h\left(x\right).$$

Using Lemma 1, we obtain

$$\left(f_{0}\left(x\right)+vf_{1}\left(x\right)\right)|\frac{x^{n_{2}}-1}{g_{1}\left(x\right)}l_{1}\left(x\right)h\left(x\right)\;\left(\mathrm{mod}\;x^{n_{1}}-1\right),$$

and hence there exists q(x)∈Re[x] such that

$$k_{2}\left(x\right)h\left(x\right)\equiv q\left(x\right)\left(f_{0}\left(x\right)+vf_{1}\left(x\right)\right)\;\left(\mathrm{mod}\;x^{n_{1}}-1\right).$$

Substituting this into Equation (3), we get

$${\left[\left(k_{1}\left(x\right)+q\left(x\right)\right)\left(f_{0}\left(x\right)+vf_{1}\left(x\right)\right)\right]}_{\left(x^{n_{1}}-1\right)}=x^{n_{1}-1-\deg\left(l\left(x\right)\right)}l^{*}\left(x\right),$$

which implies

$$u^{r}\left(x\right)=\left(x^{n_{1}-1-\deg\left(l\left(x\right)\right)}l^{*}\left(x\right);0\right)=\left(k_{1}\left(x\right)+q\left(x\right)\right)*\left(f_{0}\left(x\right)+vf_{1}\left(x\right);0\right)\in I,$$

contradicting the assumption that $u^{r}\left(x\right)\notin I.$

**Case** **2.** When $k_{2}\left(x\right)=v\frac{x^{n_{2}}-1}{g_{0}\left(x\right)}l_{2}\left(x\right)$. Then, we have

$$k_{2}\left(x\right)h\left(x\right)=v\frac{x^{n_{2}}-1}{g_{0}\left(x\right)}l_{2}\left(x\right)h\left(x\right).$$

By Lemma 1, it follows that

$$\left(f_{0}\left(x\right)+vf_{1}\left(x\right)\right)|v\frac{x^{n_{2}}-1}{g_{0}\left(x\right)}l_{2}\left(x\right)h\left(x\right),$$

and using the similar arguments as that of Case 1, we conclude that $u^{r}\left(x\right)\in I$, again leading to a contradiction. Thus, $I=\langle \left(f_{0}\left(x\right)+vf_{1}\left(x\right);0\right)\rangle$ is a reversible code. Consequently, $\zeta_{n_{1}}\left(I\right)=\langle f_{0}\left(x\right)+vf_{1}\left(x\right)\rangle$ is a reversible cyclic code of length $n_{1}$ over Re. In accordance with Theorem 2, the polynomials $f_{0}\left(x\right)$ and $f_{1}\left(x\right)$ are self-reciprocal. □

In Theorem 3, we have established the necessary conditions that the polynomials $f_{0}\left(x\right)$, $f_{1}\left(x\right)$, $g_{0}\left(x\right)$, and $g_{1}\left(x\right)$ satisfy for a code $C=\langle \left(f_{0}\left(x\right)+vf_{1}\left(x\right);0\right),\left(h\left(x\right);g_{0}\left(x\right)+vg_{1}\left(x\right)\right)\rangle$ to be reversible, where the lengths $n_{1}$ and $n_{2}$ are assumed to be odd positive integers. In the next theorem, the specific situation where $n_{2}=\left(2n_{3}+1\right)n_{1}$ for all non-negative integers $n_{3}$, we investigate the requirement that the polynomial h(x) for *C* to be reversible.

**Theorem** **4.**  Let $C=\langle \left(f_{0}\left(x\right)+vf_{1}\left(x\right);0\right),\left(h\left(x\right);g_{0}\left(x\right)+vg_{1}\left(x\right)\right)\rangle$ be a double cyclic code of length $\left(n_{1},n_{2}=\left(2n_{3}+1\right)n_{1}\right)$ over the ring Re, where the criteria in Theorem 1 are satisfied by the polynomials $f_{0}\left(x\right)$, $f_{1}\left(x\right)$, $g_{0}\left(x\right)$, $g_{1}\left(x\right)$, h(x) and $n_{3}\in Z^{+}\cup \left\{0\right\}$. Assume that $\deg\left(g_{0}\left(x\right)+vg_{1}\left(x\right)\right)=2n_{3}n_{1}+\deg\left(h\left(x\right)\right)$. If C is reversible code, then $\left(f_{0}\left(x\right)+vf_{1}\left(x\right)\right)$ divides $\left(l^{*}\left(x\right)+h\left(x\right)+\left(1+x^{i}\right)va\left(x\right)g_{1}\left(x\right)h\left(x\right)+\left(1+x^{i}\right)b\left(x\right)\frac{x^{n_{2}}-1}{g_{0}\left(x\right)}h\left(x\right)\right)$ in Rn1e, where $a\left(x\right),b\left(x\right)\in F_{4^{e}}\left[x\right]$ such that $a\left(x\right)g_{0}\left(x\right)+b\left(x\right)\frac{x^{n_{2}}-1}{g_{0}\left(x\right)}=1$ and $i=\deg\left(g_{0}\left(x\right)\right)-\deg\left(g_{1}\left(x\right)\right)$.

**Proof.**  Assume that *C* is a reversible code and let $n_{2}=\left(2n_{3}+1\right)n_{1}$, where $n_{3}\in Z^{+}\cup \left\{0\right\}$. The polynomials $f_{0}\left(x\right)$, $f_{1}\left(x\right)$, $g_{0}\left(x\right)$, and $g_{1}\left(x\right)$ are self-reciprocal according to Theorem 3. Since $(h\left(x\right);g_{0}\left(x\right)+vg_{1}\left(x\right))\in C$, it follows that

$${\left(h\left(x\right);g_{0}\left(x\right)+vg_{1}\left(x\right)\right)}^{r}=\left(x^{n_{1}-1-\deg\left(h\left(x\right)\right)}l^{*}\left(x\right);x^{n_{2}-1-\deg\left(g_{0}\left(x\right)+vg_{1}\left(x\right)\right)}{\left(g_{0}\left(x\right)+vg_{1}\left(x\right)\right)}^{*}\right)\in C.$$

Which implies

$$\begin{array}{c} \left(h^{*}\left(x\right);{\left(g_{0}\left(x\right)+vg_{1}\left(x\right)\right)}^{*}\right)\\ =\;x^{2n_{3}n_{1}+\deg\left(h\left(x\right)\right)+1}*\left(x^{n_{1}-1-\deg\left(h\left(x\right)\right)}h^{*}\left(x\right);x^{n_{2}-1-\deg\left(g_{0}\left(x\right)+vg_{1}\left(x\right)\right)}{\left(g_{0}\left(x\right)+vg_{1}\left(x\right)\right)}^{*}\right)\in C. \end{array}$$

Hence, there exist some k1(x),k2(x)∈Re[x] such that

$$\left(h^{*}\left(x\right);{\left(g_{0}\left(x\right)+vg_{1}\left(x\right)\right)}^{*}\right)=k_{1}\left(x\right)*\left(f_{0}\left(x\right)+vf_{1}\left(x\right);0\right)+k_{2}\left(x\right)*\left(h\left(x\right);g_{0}\left(x\right)+vg_{1}\left(x\right)\right).$$

Consequently, we have

(5) $${\left[k_{1}\left(x\right)\left(f_{0}\left(x\right)+vf_{1}\left(x\right)\right)+k_{2}\left(x\right)h\left(x\right)\right]}_{\left(x^{n_{1}}-1\right)}=h^{*}\left(x\right)$$

(6) $${\left[k_{2}\left(x\right)\left(g_{0}\left(x\right)+vg_{1}\left(x\right)\right)\right]}_{\left(x^{n_{2}}-1\right)}=g_{0}\left(x\right)+vx^{i}g_{1}\left(x\right),$$

where $i=\deg\left(g_{0}\left(x\right)\right)-\deg\left(g_{1}\left(x\right)\right)$. Consider the following congruences:

(7) $${\left[v\left(g_{0}\left(x\right)+vg_{1}\left(x\right)\right)\right]}_{\left(x^{n_{2}}-1\right)}=vg_{0}\left(x\right)\;\mathrm{and}$$

(8) $${\left[\frac{x^{n_{2}}-1}{g_{0}\left(x\right)}\left(g_{0}\left(x\right)+vg_{1}\left(x\right)\right)\right]}_{\left(x^{n_{2}}-1\right)}=\frac{x^{n_{2}}-1}{g_{0}\left(x\right)}vg_{1}\left(x\right).$$

Since $\gcd(g_{0}\left(x\right),\frac{x^{n_{2}}-1}{g_{0}\left(x\right)})=1$, so we have $a\left(x\right)g_{0}\left(x\right)+b\left(x\right)\frac{x^{n_{2}}-1}{g_{0}\left(x\right)}=1,$ for some polynomials $a\left(x\right),b\left(x\right)\in F_{4^{e}}\left[x\right]$. Multiplying on both sides by $vg_{1}\left(x\right)$, we obtain

$$vg_{1}\left(x\right)=vg_{1}\left(x\right)\cdot \left(a\left(x\right)g_{0}\left(x\right)+b\left(x\right)\frac{x^{n_{2}}-1}{g_{0}\left(x\right)}\right)=vg_{1}\left(x\right)a\left(x\right)g_{0}\left(x\right)+vg_{1}\left(x\right)b\left(x\right)\frac{x^{n_{2}}-1}{g_{0}\left(x\right)}.$$

Then, using Equations (7) and (8), we have

$${\left[\left(va\left(x\right)g_{1}\left(x\right)+b\left(x\right)\frac{x^{n_{2}}-1}{g_{0}\left(x\right)}\right)\left(g_{0}\left(x\right)+vg_{1}\left(x\right)\right)\right]}_{\left(x^{n_{2}}-1\right)}=vg_{1}\left(x\right).$$

Therefore,

$$vx^{i}g_{1}\left(x\right)\equiv x^{i}\left(va\left(x\right)g_{1}\left(x\right)+b\left(x\right)\frac{x^{n_{2}}-1}{g_{0}\left(x\right)}\right)\left(g_{0}\left(x\right)+vg_{1}\left(x\right)\right)\;\left(\mathrm{mod}\;x^{n_{2}}-1\right),$$

and

$$g_{0}\left(x\right)=g_{0}\left(x\right)+vg_{1}\left(x\right)+vg_{1}\left(x\right)$$

$$\equiv \left(1+va\left(x\right)g_{1}\left(x\right)+b\left(x\right)\frac{x^{n_{2}}-1}{g_{0}\left(x\right)}\right)\left(g_{0}\left(x\right)+vg_{1}\left(x\right)\right)\;\left(\mathrm{mod}\;x^{n_{2}}-1\right).$$

Hence,

$$g_{0}\left(x\right)+vx^{i}g_{1}\left(x\right)={\left[\left(1+\left(1+x^{i}\right)va\left(x\right)g_{1}\left(x\right)+\left(1+x^{i}\right)b\left(x\right)\frac{x^{n_{2}}-1}{g_{0}\left(x\right)}\right)\left(g_{0}\left(x\right)+vg_{1}\left(x\right)\right)\right]}_{\left(x^{n_{2}}-1\right)}.$$

Applying Equation (6), we get

$\left(k_{2}\left(x\right)-\left(1+\left(1+x^{i}\right)va\left(x\right)g_{1}\left(x\right)+\left(1+x^{i}\right)b\left(x\right)\frac{x^{n_{2}}-1}{g_{0}\left(x\right)}\right)\right)\left(g_{0}\left(x\right)+vg_{1}\left(x\right)\right)\equiv 0\;\left(\mathrm{mod}\;x^{n_{2}}-1\right).$ This implies that the expression

$$k_{2}\left(x\right)-\left(1+\left(1+x^{i}\right)va\left(x\right)g_{1}\left(x\right)+\left(1+x^{i}\right)b\left(x\right)\frac{x^{n_{2}}-1}{g_{0}\left(x\right)}\right)$$

is either 0 or of the form $\frac{x^{n_{2}}-1}{g_{1}\left(x\right)}\left(l_{1}\left(x\right)\right)$, or $v\frac{x^{n_{2}}-1}{g_{0}\left(x\right)}\left(l_{2}\left(x\right)\right)$, where l1(x),l2(x)∈Re[x].

**Case** **1.** If $k_{2}\left(x\right)-\left(1+\left(1+x^{i}\right)va\left(x\right)g_{1}\left(x\right)+\left(1+x^{i}\right)b\left(x\right)\frac{x^{n_{2}}-1}{g_{0}\left(x\right)}\right)=0$, then

$$k_{2}\left(x\right)=\left(1+\left(1+x^{i}\right)va\left(x\right)g_{1}\left(x\right)+\left(1+x^{i}\right)b\left(x\right)\frac{x^{n_{2}}-1}{g_{0}\left(x\right)}\right).$$

From Equation (5), we have $h^{*}\left(x\right)=[k_{1}\left(x\right)\left(f_{0}\left(x\right)+vf_{1}\left(x\right)\right)+h\left(x\right)+\left(1+x^{i}\right)va\left(x\right)g_{1}\left(x\right)h\left(x\right)+\left(1+x^{i}\right)b\left(x\right)\frac{x^{n_{2}}-1}{g_{0}\left(x\right)}h\left(x\right){]}_{\left(x^{n_{1}}-1\right)}.$ This yields

$$\begin{array}{cc} \; & h^{*}\left(x\right)+h\left(x\right)+\left(1+x^{i}\right)va\left(x\right)g_{1}\left(x\right)h\left(x\right)+\left(1+x^{i}\right)b\left(x\right)\frac{x^{n_{2}}-1}{g_{0}\left(x\right)}h\left(x\right)\\ \; & \;\equiv k_{1}\left(x\right)\left(f_{0}\left(x\right)+vf_{1}\left(x\right)\right)\;\left(\mathrm{mod}\;x^{n_{1}}-1\right). \end{array}$$

Hence,

$$\left(f_{0}\left(x\right)+vf_{1}\left(x\right)\right)|\left(h^{*}\left(x\right)+h\left(x\right)+\left(1+x^{i}\right)va\left(x\right)g_{1}\left(x\right)h\left(x\right)+\left(1+x^{i}\right)b\left(x\right)\frac{x^{n_{2}}-1}{g_{0}\left(x\right)}h\left(x\right)\right)$$

in Rn1e.

**Case** **2.** When $k_{2}\left(x\right)-\left(1+\left(1+x^{i}\right)va\left(x\right)g_{1}\left(x\right)+\left(1+x^{i}\right)b\left(x\right)\frac{x^{n_{2}}-1}{g_{0}\left(x\right)}\right)=\frac{x^{n_{2}}-1}{g_{1}\left(x\right)}l_{1}\left(x\right)$, then we have

$$k_{2}\left(x\right)=\frac{x^{n_{2}}-1}{g_{1}\left(x\right)}l_{1}\left(x\right)+\left(1+\left(1+x^{i}\right)va\left(x\right)g_{1}\left(x\right)+\left(1+x^{i}\right)b\left(x\right)\frac{x^{n_{2}}-1}{g_{0}\left(x\right)}\right).$$

Multiplying on both sides by h(x), we obtain

$$k_{2}\left(x\right)h\left(x\right)=\frac{x^{n_{2}}-1}{g_{1}\left(x\right)}l_{1}\left(x\right)h\left(x\right)+\left(1+\left(1+x^{i}\right)va\left(x\right)g_{1}\left(x\right)+\left(1+x^{i}\right)b\left(x\right)\frac{x^{n_{2}}-1}{g_{0}\left(x\right)}\right)h\left(x\right).$$

It is evident from the Lemma 1, there exists some q(x)∈Re[x] such that

$$\frac{x^{n_{2}}-1}{g_{1}\left(x\right)}l_{1}\left(x\right)h\left(x\right)\equiv q\left(x\right)\left(f_{0}\left(x\right)+vf_{1}\left(x\right)\right)\;\left(\mathrm{mod}\;x^{n_{1}}-1\right).$$

Thus, according to Equation (5), we have

$$\begin{array}{cc} \; & h^{*}\left(x\right)+h\left(x\right)+\left(1+x^{i}\right)va\left(x\right)g_{1}\left(x\right)h\left(x\right)+\left(1+x^{i}\right)b\left(x\right)\frac{x^{n_{2}}-1}{g_{0}\left(x\right)}h\left(x\right)\\ \; & \;\equiv \left(k_{1}\left(x\right)+q\left(x\right)\right)\left(f_{0}\left(x\right)+vf_{1}\left(x\right)\right)\;\left(\mathrm{mod}\;x^{n_{1}}-1\right). \end{array}$$

Hence,

(f0(x)+vf1(x))∣h*(x)+h(x)+(1+xi)va(x)g1(x)h(x)+(1+xi)b(x)xn2−1g0(x)h(x) in Rn1e.

**Case** **3.** Suppose $k_{2}\left(x\right)-\left(1+\left(1+x^{i}\right)va\left(x\right)g_{1}\left(x\right)+\left(1+x^{i}\right)b\left(x\right)\frac{x^{n_{2}}-1}{g_{0}\left(x\right)}\right)=v\frac{x^{n_{2}}-1}{g_{0}\left(x\right)}l_{2}\left(x\right).$ Then,

$$k_{2}\left(x\right)=v\frac{x^{n_{2}}-1}{g_{0}\left(x\right)}l_{2}\left(x\right)+\left(1+\left(1+x^{i}\right)va\left(x\right)g_{1}\left(x\right)+\left(1+x^{i}\right)b\left(x\right)\frac{x^{n_{2}}-1}{g_{0}\left(x\right)}\right).$$

Therefore,

$$k_{2}\left(x\right)h\left(x\right)=v\frac{x^{n_{2}}-1}{g_{0}\left(x\right)}l_{2}\left(x\right)h\left(x\right)+\left(1+\left(1+x^{i}\right)va\left(x\right)g_{1}\left(x\right)+\left(1+x^{i}\right)b\left(x\right)\frac{x^{n_{2}}-1}{g_{0}\left(x\right)}\right)h\left(x\right).$$

By Lemma 1, there exists some q(x)∈Re[x] satisfying

$$v\frac{x^{n_{2}}-1}{g_{0}\left(x\right)}l_{2}\left(x\right)h\left(x\right)\equiv q\left(x\right)\left(f_{0}\left(x\right)+vf_{1}\left(x\right)\right)\;\left(\mathrm{mod}\;x^{n_{1}}-1\right).$$

Analogous to Case 2 mentioned earlier, we derive that

$$\left(f_{0}\left(x\right)+vf_{1}\left(x\right)\right)|\left(h^{*}\left(x\right)+h\left(x\right)+\left(1+x^{i}\right)va\left(x\right)g_{1}\left(x\right)h\left(x\right)+\left(1+x^{i}\right)b\left(x\right)\frac{x^{n_{2}}-1}{g_{0}\left(x\right)}h\left(x\right)\right)$$

in Rn1e. Combining all of the three cases, it follows that

$$\left(f_{0}\left(x\right)+vf_{1}\left(x\right)\right)|\left(h^{*}\left(x\right)+h\left(x\right)+\left(1+x^{i}\right)va\left(x\right)g_{1}\left(x\right)h\left(x\right)+\left(1+x^{i}\right)b\left(x\right)\frac{x^{n_{2}}-1}{g_{0}\left(x\right)}h\left(x\right)\right)$$

in Rn1e, where $a\left(x\right),b\left(x\right)\in F_{4^{e}}\left[x\right]$ such that $a\left(x\right)g_{0}\left(x\right)+b\left(x\right)\frac{x^{n_{2}}-1}{g_{0}\left(x\right)}=1$ and $i=\deg\left(g_{0}\left(x\right)\right)-\deg\left(g_{1}\left(x\right)\right)$. □

As a consequence of Theorems 3 and 4, we arrive at the following result:

**Theorem** **5.**  Let $C=\langle \left(f_{0}\left(x\right)+vf_{1}\left(x\right);0\right),\left(h\left(x\right);g_{0}\left(x\right)+vg_{1}\left(x\right)\right)\rangle$ be a double cyclic code of length $\left(n_{1},n_{2}=\left(2n_{3}+1\right)n_{1}\right)$ over the ring Re, where the criteria in Theorem 1 are satisfied by the polynomials $f_{0}\left(x\right)$, $f_{1}\left(x\right)$, $g_{0}\left(x\right)$, $g_{1}\left(x\right),h\left(x\right)$, and $n_{3}\in Z^{+}$. Assume that $\deg\left(g_{0}\left(x\right)+vg_{1}\left(x\right)\right)=2n_{3}n_{1}+\deg\left(h\left(x\right)\right)$. Then, the code C is reversible if $f_{0}\left(x\right),f_{1}\left(x\right),g_{0}\left(x\right),g_{1}\left(x\right)$ are self-reciprocal, and $\left(f_{0}\left(x\right)+vf_{1}\left(x\right)\right)|\left(h^{*}\left(x\right)+h\left(x\right)+\left(1+x^{i}\right)va\left(x\right)g_{1}\left(x\right)h\left(x\right)+\left(1+x^{i}\right)b\left(x\right)\frac{x^{n_{2}}-1}{g_{0}\left(x\right)}h\left(x\right)\right)$ in Rn1e, where $a\left(x\right),b\left(x\right)\in F_{4^{e}}\left[x\right]$ satisfying $a\left(x\right)g_{0}\left(x\right)+b\left(x\right)\frac{x^{n_{2}}-1}{g_{0}\left(x\right)}=1$ and $i=\deg\left(g_{0}\left(x\right)\right)-\deg\left(g_{1}\left(x\right)\right)$.

**Proof.**  Assume that *C* is reversible code. The intended outcome is directly obtained by using Theorems 3 and 4.

Conversely, suppose that $f_{0}\left(x\right),f_{1}\left(x\right),g_{0}\left(x\right),g_{1}\left(x\right)$ are self-reciprocal polynomials and

$$\left(f_{0}\left(x\right)+vf_{1}\left(x\right)\right)|\left(h^{*}\left(x\right)+h\left(x\right)+\left(1+x^{i}\right)va\left(x\right)g_{1}\left(x\right)h\left(x\right)+\left(1+x^{i}\right)b\left(x\right)\frac{x^{n_{2}}-1}{g_{0}\left(x\right)}h\left(x\right)\right)$$

in Rn1e, where $a\left(x\right),b\left(x\right)\in F_{4^{e}}\left[x\right]$ satisfy the identity $a\left(x\right)g_{0}\left(x\right)+b\left(x\right)\frac{x^{n_{2}}-1}{g_{0}\left(x\right)}=1$ and $i=\deg\left(g_{0}\left(x\right)\right)-\deg\left(g_{1}\left(x\right)\right)$. Given that $a\left(x\right)g_{0}\left(x\right)+b\left(x\right)\frac{x^{n_{2}}-1}{g_{0}\left(x\right)}=1.$ Therefore, it follows that

$${\left[\left(1+\left(1+x^{i}\right)va\left(x\right)g_{1}\left(x\right)+\left(1+x^{i}\right)b\left(x\right)\frac{x^{n_{2}}-1}{g_{0}\left(x\right)}\right)\left(g_{0}\left(x\right)+vg_{1}\left(x\right)\right)\right]}_{\left(x^{n_{2}}-1\right)}=g_{0}\left(x\right)+vx^{i}g_{1}\left(x\right).$$

Since

$$\left(f_{0}\left(x\right)+vf_{1}\left(x\right)\right)|\left(h^{*}\left(x\right)+h\left(x\right)+\left(1+x^{i}\right)va\left(x\right)g_{1}\left(x\right)h\left(x\right)+\left(1+x^{i}\right)b\left(x\right)\frac{x^{n_{2}}-1}{g_{0}\left(x\right)}h\left(x\right)\right)$$

in Rn1e, there exists k(x)∈Re[x] such that

$$\begin{array}{cc} \; & h^{*}\left(x\right)+h\left(x\right)+\left(1+x^{i}\right)va\left(x\right)g_{1}\left(x\right)h\left(x\right)+\left(1+x^{i}\right)b\left(x\right)\frac{x^{n_{2}}-1}{g_{0}\left(x\right)}h\left(x\right)\\ \; & \;\equiv k\left(x\right)\left(f_{0}\left(x\right)+vf_{1}\left(x\right)\right)\;\left(\mathrm{mod}\;x^{n_{1}}-1\right). \end{array}$$

Hence, we can express the pair $\left(h^{*}\left(x\right);{\left(g_{0}\left(x\right)+vg_{1}\left(x\right)\right)}^{*}\right)$ as

$$\begin{array}{cc} \; & \left(h^{*}\left(x\right);{\left(g_{0}\left(x\right)+vg_{1}\left(x\right)\right)}^{*}\right)\\ \; & =(\left(f_{0}\left(x\right)+vf_{1}\left(x\right)\right)k\left(x\right)+h\left(x\right)+\left(1+x^{i}\right)va\left(x\right)g_{1}\left(x\right)h\left(x\right)+\left(1+x^{i}\right)b\left(x\right)\frac{x^{n_{2}}-1}{g_{0}\left(x\right)}h\left(x\right);(g_{0}\left(x\right)+\\ \; & \;vg_{1}\left(x\right))+\left(1+x^{i}\right)va\left(x\right)g_{1}\left(x\right)\left(g_{0}\left(x\right)+vg_{1}\left(x\right)\right)+\left(1+x^{i}\right)b\left(x\right)\frac{x^{n_{2}}-1}{g_{0}\left(x\right)}\left(g_{0}\left(x\right)+vg_{1}\left(x\right)\right))\\ \; & =k\left(x\right)*\left(f_{0}\left(x\right)+vf_{1}\left(x\right);0\right)+\left(h\left(x\right);g_{0}\left(x\right)+vg_{1}\left(x\right)\right)+\left(\left(1+x^{i}\right)va\left(x\right)g_{1}\left(x\right)\right)*(h\left(x\right);g_{0}\left(x\right)+\\ \; & \;vg_{1}\left(x\right))+\left(\left(1+x^{i}\right)b\left(x\right)\frac{x^{n_{2}}-1}{g_{0}\left(x\right)}\right)*\left(h\left(x\right);g_{0}\left(x\right)+vg_{1}\left(x\right)\right). \end{array}$$

This implies that $(h^{*}\left(x\right);{\left(g_{0}\left(x\right)+vg_{1}\left(x\right)\right)}^{*})\in C$. Since $\gcd(f_{0}\left(x\right),\frac{x^{n_{1}}-1}{f_{0}\left(x\right)})=1$, so we have $a^{\prime }\left(x\right)f_{0}\left(x\right)+b^{\prime }\left(x\right)\frac{x^{n_{1}}-1}{f_{0}\left(x\right)}=1,$ for some $a^{\prime }\left(x\right),b^{\prime }\left(x\right)\in F_{4^{e}}\left[x\right]$. Therefore, we obtain

$${\left[1+\left(1+x^{j}\right)va^{\prime }\left(x\right)f_{1}\left(x\right)+\left(1+x^{j}\right)b^{\prime }\left(x\right)\frac{x^{n_{1}}-1}{f_{0}\left(x\right)})\left(f_{0}\left(x\right)+vf_{1}\left(x\right)\right)\right]}_{\left(x^{n_{1}}-1\right)}=f_{0}\left(x\right)+vx^{j}f_{1}\left(x\right),$$

where $j=\deg\left(f_{0}\left(x\right)\right)-\deg\left(f_{1}\left(x\right)\right)$. Hence,

$$\begin{array}{cc} \; & \left({\left(f_{0}\left(x\right)+vf_{1}\left(x\right)\right)}^{*};0\right)\\ \; & =(f_{0}\left(x\right)+vx^{j}f_{1}\left(x\right);0)\\ \; & =\left(1+\left(1+x^{j}\right)va^{\prime }\left(x\right)f_{1}\left(x\right)+\left(1+x^{j}\right)b^{\prime }\left(x\right)\frac{x^{n_{1}}-1}{f_{0}\left(x\right)}\right)*\left(f_{0}\left(x\right)+vf_{1}\left(x\right);0\right)\in C. \end{array}$$

This implies that ${\left(f_{0}\left(x\right)+vf_{1}\left(x\right)\right)}^{*};0)\in C$. Next, we have to show that *C* is reversible code. Let u(x)∈C be any codeword. Then, there exist some k1(x),k2(x)∈Re[x] such that

$$u\left(x\right)=k_{1}\left(x\right)*\left(f_{0}\left(x\right)+vf_{1}\left(x\right);0\right)+k_{2}\left(x\right)*\left(h\left(x\right);g_{0}\left(x\right)+vg_{1}\left(x\right)\right).$$

Consider the reverse of u(x), we obtain

$$u^{r}\left(x\right)={\left(k_{1}\left(x\right)\left(f_{0}\left(x\right)+vf_{1}\left(x\right)\right)+k_{2}\left(x\right)h\left(x\right);k_{2}\left(x\right)\left(g_{0}\left(x\right)+vg_{1}\left(x\right)\right)\right)}^{r}.$$

Using Proposition 6, we get

$$u^{r}\left(x\right)={\left(k_{1}\left(x\right)\left(f_{0}\left(x\right)+vf_{1}\left(x\right)\right);0\right)}^{r}+{\left(k_{2}\left(x\right)h\left(x\right);k_{2}\left(x\right)\left(g_{0}\left(x\right)+vg_{1}\left(x\right)\right)\right)}^{r}.$$

Let k1(x)=a0+a1x+…+asxs∈Re[x], where *s* is a non-negative integer. Then, we consider

$$\begin{array}{ccc} {\left(k_{1}\left(x\right)\left(f_{0}\left(x\right)+vf_{1}\left(x\right)\right);0\right)}^{r} & = & {\left(\sum_{i=0}^{s}a_{i}x^{i}\left(f_{0}\left(x\right)+vf_{1}\left(x\right)\right);0\right)}^{r}\\ & = & \sum_{i=0}^{s}a_{i}{\left(x^{i}\left(f_{0}\left(x\right)+vf_{1}\left(x\right)\right);0\right)}^{r}. \end{array}$$

From Proposition 6, it follows that there exists $p_{i}\in Z^{+}\cup \left\{0\right\}$ such that

$${\left(x^{i}\left(f_{0}\left(x\right)+vf_{1}\left(x\right)\right);0\right)}^{r}=x^{(p_{i}+1)a-1-j}*\left({\left(f_{0}\left(x\right)+vf_{1}\left(x\right)\right)}^{*};0\right),$$

where $j=\deg(x^{i}\left(f_{0}\left(x\right)+vf_{1}\left(x\right)\right))$ and 0≤i≤s. Therefore, ${\left(k_{1}\left(x\right)\left(f_{0}\left(x\right)+vf_{1}\left(x\right)\right);0\right)}^{r}\in C$.

Assume that k2(x)=b0+b1x+…+btxt∈Re[x] in which *t* denotes a non-negative integer. Now, we analyze

$$\begin{array}{ccc} {\left(k_{2}\left(x\right)h\left(x\right);k_{2}\left(x\right)\left(g_{0}\left(x\right)+vg_{1}\left(x\right)\right)\right)}^{r} & = & {\left(\sum_{j=0}^{t}b_{j}x^{j}h\left(x\right);\sum_{j=0}^{t}b_{j}x^{j}\left(g_{0}\left(x\right)+vg_{1}\left(x\right)\right)\right)}^{r}\\ & = & \sum_{j=0}^{t}b_{j}{\left(x^{j}h\left(x\right);x^{j}\left(g_{0}\left(x\right)+vg_{1}\left(x\right)\right)\right)}^{r}. \end{array}$$

According to the Corollary 2, we have

$${\left(x^{j}h\left(x\right);x^{j}\left(g_{0}\left(x\right)+vg_{1}\left(x\right)\right)\right)}^{r}=x^{\left(q_{j}\left(2n_{3}+1\right)+1\right)n_{1}-1-\deg\left(x^{j}h\left(x\right)\right)}*\left(h^{*}\left(x\right);{\left(g_{0}\left(x\right)+vg_{1}\left(x\right)\right)}^{*}\right),$$

for some $q_{j}\in Z^{+}\cup \left\{0\right\}$ with 0≤j≤t. Thus, we obtain ${\left(k_{2}\left(x\right)h\left(x\right);k_{2}\left(x\right)\left(g_{0}\left(x\right)+vg_{1}\left(x\right)\right)\right)}^{r}\in C$. Therefore,

$$v^{r}\left(x\right)={\left(k_{1}\left(x\right)\left(f_{0}\left(x\right)+vf_{1}\left(x\right)\right);0\right)}^{r}+{\left(k_{2}\left(x\right)h\left(x\right);k_{2}\left(x\right)\left(g_{0}\left(x\right)+vg_{1}\left(x\right)\right)\right)}^{r}\in C.$$

Hence, *C* is reversible. □

As an immediate consequences of Theorem 5, we obtain the following corollary:

**Corollary** **3.**  *Let $C=\langle \left(f_{0}\left(x\right)+vf_{1}\left(x\right);0\right),\left(h\left(x\right);g_{0}\left(x\right)+vg_{1}\left(x\right)\right)\rangle$ be a double cyclic code of length $\left(n_{1},n_{2}=\left(2n_{3}+1\right)n_{1}\right)$ over Re, where the criteria in Theorem 1 are satisfied by the polynomials $f_{0}\left(x\right)$, $f_{1}\left(x\right)$, $g_{0}\left(x\right)$, $g_{1}\left(x\right)$, h(x), and $n_{3}\in Z^{+}\cup \{0\}$. Assume that $\deg\left(g_{0}\left(x\right)\right)=\deg\left(g_{1}\left(x\right)\right)=2n_{3}n_{1}+\deg\left(h\left(x\right)\right)$. Then, C is reversible code iff $f_{0}\left(x\right),f_{1}\left(x\right),g_{0}\left(x\right),g_{1}\left(x\right),$ and h(x) are self-reciprocal polynomials.*

### 3.2. Reversible-Complement DNA Codes over Re

This subsection establishes the conditions under which double cyclic codes become reversible-complement codes and provides illustrative examples of DNA codes obtained from them.

**Lemma** **3.**  *Let C be a double cyclic code of length $\left(n_{1},n_{2}\right)$ over the ring Re. If the code C is reversible-complement. Then, it contains the following codeword*

$$\left(\underset{n_{1}}{\underset{︸}{v,v,v,\ldots ,v}};\underset{n_{2}}{\underset{︸}{v,v,v,\ldots ,v}}\right):=vI\left(x\right)=\left(v+vx+vx^{2}+\ldots +vx^{n_{1}-1};v+vx+vx^{2}+\ldots +vx^{n_{2}-1}\right).$$

**Proof.**  Let $z^{r}$ be the coordinate-reversal of z on Rn1,n2e, and let the DNA complement on Re be the map $a\mapsto \bar{a}$ determined by $a+\bar{a}=v$ (hence $\bar{a}=v-a$ componentwise). Define the reversible-complement operator *T* on Rn1,n2e by

(9) $$T\left(z\right)=\bar{z^{r}}=v1-z^{r},$$

where z∈Rn1,n2e, $1=\left(\underset{n_{1}}{\underset{︸}{1,1,1,\ldots ,1}};\underset{n_{2}}{\underset{︸}{1,1,1,\ldots ,1}}\right),$ and subtraction is componentwise in Re. Thus *T* is a map of the form “translation by v1” composed with the R-linear permutation $z^{r}$ of z. Since $0=\left(\underset{n_{1}}{\underset{︸}{0,0,\ldots ,0}};\underset{n_{2}}{\underset{︸}{0,0,\ldots ,0}}\right)\in C$, so taking z=0 in Equation (9), we obtain

$$T\left(0\right)\;=\;v1-0^{r}=v1-0\;=\;v1\in C.$$

In polynomial form for double cyclic code *C*, v1 corresponds exactly to

$$\begin{array}{ccc} v1 & = & v\left(\underset{n_{1}}{\underset{︸}{1,1,1,\ldots ,1}};\underset{n_{2}}{\underset{︸}{1,1,1,\ldots ,1}}\right)=\left(\underset{n_{1}}{\underset{︸}{v,v,v,\ldots ,v}};\underset{n_{2}}{\underset{︸}{v,v,v,\ldots ,v}}\right)\\ & = & \left(v+vx+vx^{2}\ldots +vx^{n_{1}-1};\;v+vx+vx^{2}+\ldots +vx^{n_{2}-1}\right)=vI\left(x\right). \end{array}$$

Hence, vI(x)∈C. □

**Theorem** **6.**  *Let C be a double cyclic code of length $\left(n_{1},n_{2}\right)$ over Re. Then, the code C is reversible-complement if*

*(i)*  *C is reversible, and*
*(ii)*  *$\left(\underset{n_{1}}{\underset{︸}{v,v,v,\ldots ,v}};\underset{n_{2}}{\underset{︸}{v,v,v,\ldots ,v}}\right)\in C$.*

**Proof.**  We begin the proof by invoking Equation (9) established in the preceding theorem, since the characteristic of the ring Re is 2, we have

(10) $$T\left(z\right)=v1+z^{r}.$$

If *C* is reversible-complement, then T(C)⊆C. In particular, T(0)=v1∈C. Thus, $\left(\underset{n_{1}}{\underset{︸}{v,v,v,\ldots ,v}};\underset{n_{2}}{\underset{︸}{v,v,v,\ldots ,v}}\right)\in C$. Now, for any z∈C, we have T(z)∈C, hence $z^{r}=T\left(z\right)+v1\in C$ (since *C* linear), so *C* is reversible.

Conversely, assume *C* is reversible and v1∈C. Then for every z∈C, $z^{r}\in C$; therefore $T\left(z\right)=v1+z^{r}\in C$, i.e., the code *C* is reversible-complement. This completes the proof. □

By combining the results from Section 3.1 with Theorem 6, we directly obtain the following results:

**Proposition** **7.**  *Let C be a double cyclic code of length $\left(n_{1},n_{2}\right)$ over Re and assume that $C=\langle \left(f_{0}\left(x\right)+vf_{1}\left(x\right);0\right),\left(0;g_{0}\left(x\right)+vg_{1}\left(x\right)\right)\rangle$ is a separable code, where the criteria in Theorem 1 are satisfied by the polynomials $f_{0}\left(x\right)$, $f_{1}\left(x\right)$, $g_{0}\left(x\right)$, and $g_{1}\left(x\right)$. Then, the code C is reversible-complement iff $f_{0}\left(x\right),f_{1}\left(x\right),g_{0}\left(x\right)$, and $g_{1}\left(x\right)$ are self-reciprocal polynomials and vI(x)∈C.*

**Theorem** **7.**  *Let $C=\langle \left(f_{0}\left(x\right)+vf_{1}\left(x\right);0\right),\left(h\left(x\right);g_{0}\left(x\right)+vg_{1}\left(x\right)\right)\rangle$ be a double cyclic code of length $\left(n_{1},n_{2}=\left(2n_{3}+1\right)n_{1}\right)$ over the ring Re, where the criteria in Theorem 1 are satisfied by the polynomials $f_{0}\left(x\right)$, $f_{1}\left(x\right)$, $g_{0}\left(x\right)$, $g_{1}\left(x\right)$, h(x), and $n_{3}\in Z^{+}\cup \left\{0\right\}$. Assume that $\deg\left(g_{0}\left(x\right)+vg_{1}\left(x\right)\right)=2n_{3}n_{1}+\deg\left(h\left(x\right)\right).$ Then, the code C is reversible-complement iff*

*(i)*  *vI(x)∈C,*
*(ii)*  *$f_{0}\left(x\right)$, $f_{1}\left(x\right)$, $g_{0}\left(x\right)$, and $g_{1}\left(x\right)$ are self-reciprocal polynomials,*
*(iii)*  $\left(f_{0}\left(x\right)+vf_{1}\left(x\right)\right)|\left(h^{*}\left(x\right)+h\left(x\right)+\left(1+x^{i}\right)va\left(x\right)g_{1}\left(x\right)h\left(x\right)+\left(1+x^{i}\right)b\left(x\right)\frac{x^{n_{2}}-1}{g_{0}\left(x\right)}h\left(x\right)\right)$ in Rn1e for some $a\left(x\right),b\left(x\right)\in F_{4^{e}}\left[x\right]$ satisfying $a\left(x\right)g_{0}\left(x\right)+b\left(x\right)\frac{x^{n_{2}}-1}{g_{0}\left(x\right)}=1,$ where $i=\deg\left(g_{0}\left(x\right)\right)-\deg\left(g_{1}\left(x\right)\right)$.

**Corollary** **4.**  *Let $C=\langle \left(f_{0}\left(x\right)+vf_{1}\left(x\right);0\right),\left(h\left(x\right);g_{0}\left(x\right)+vg_{1}\left(x\right)\right)\rangle$ be a double cyclic code of length $\left(n_{1},n_{2}=\left(2n_{3}+1\right)n_{1}\right)$ over the ring Re, where the criteria in Theorem 1 are satisfied by the polynomials $f_{0}\left(x\right)$, $f_{1}\left(x\right)$, $g_{0}\left(x\right)$, $g_{1}\left(x\right)$, and h(x), with $n_{3}\in Z^{+}\cup \left\{0\right\}$. Assume that $\deg\left(g_{0}\left(x\right)\right)=\deg\left(g_{1}\left(x\right)\right)=2n_{3}n_{1}+\deg\left(h\left(x\right)\right).$ Then, the code C is reversible-complement iff $f_{0}\left(x\right)$, $f_{1}\left(x\right)$, $g_{0}\left(x\right)$, $g_{1}\left(x\right)$, h(x) are self-reciprocal polynomials, and C contains vI(x).*

**Proof.**  The proof proceeds in a manner analogous to that of Corollary 3. □

### 3.3. Gray Map of Re

Let *C* be a double cyclic code of length $\left(n_{1},n_{2}\right)$ over the ring Re and S={A,T,G,C} be set of nucleotide. Recall that a Gray map $\eta :F_{4}\to S$ defined by η(0)=A, η(1)=G, η(v)=T, and η(1+v)=G. Since $F_{4^{e}}$ is a *e*-dimensional vector space over $F_{4}$, we can represent any element $x\in F_{4^{e}}$ as $x=x_{0}+x_{1}\beta +x_{2}\beta^{2}+\ldots +x_{e-1}\beta^{e-1},\;x_{i}\in F_{4},$ where β is a root of an irreducible polynomial over $F_{4}$. Now, define a map $\phi :F_{4^{e}}\to S^{e}$ by

$$\phi \left(x\right)=\phi \left(x_{0}+x_{1}\beta +x_{2}\beta^{2}+\ldots +x_{e-1}\beta^{e-1}\right)=\left(\eta \left(x_{0}\right),\eta \left(x_{1}\right),\ldots ,\eta \left(x_{e-1}\right)\right).$$

So, clearly from the above map ϕ, each element in $F_{4^{e}}$ gets mapped to a DNA sequence of length *e*. The following Gray map is similar to the classical Gray map over $F_{2}+vF_{2}$ with $v^{2}=0$ [13]. Define G:Re→F4e2 by $G\left(c_{1}+vc_{2}\right)=\left(c_{2},c_{1}+c_{2}\right),$ where $c_{1},c_{2}\in F_{4^{e}}.$ Now, we extend the Gray map G to a Gray map Φ:C⊆Rn1,n2e→Sn1+n2 by

$$\Phi \left(a_{0},a_{1},\ldots ,a_{n_{1}-1};b_{0},b_{1},\ldots ,b_{n_{2}-1}\right)=\left(\eta \left(a_{0}\right),\eta \left(a_{1}\right),\ldots ,\eta \left(a_{n_{1}-1}\right);\eta \left(b_{0}\right),\eta \left(b_{1}\right),\ldots ,\eta \left(b_{n_{2}-1}\right)\right),$$

where $(a_{0},a_{1},\ldots ,a_{n_{1}-1};b_{0},b_{1},\ldots ,b_{n_{2}-1})\in C$, ai,bj∈Re with $0\leq i\leq n_{1}-1,0\leq j\leq n_{2}-1$.

For any double cyclic code *C* of length $\left(n_{1},n_{2}\right)$ over Re, we let $\hat{C}$ be a set defined as

C^={(b;a)∈Rn2,n1e:(a;b)∈C}⊆Rn2,n1e.

Then, a DNA code $C_{DNA}$ can be obtained from $C\cup \hat{C}$, where *C* is taken to be a double cyclic DNA code of length $\left(n_{1},n_{2}\right)$ over the ring Re. To illustrate the above results, we now provide some concrete examples. The Magma computational algebra system [32] is used to complete all of the computations in the following Table 1 and Table 2. In the following examples, we present DNA correspondence $\Phi (C\cup \hat{C})$ for the double cyclic codes *C* over R1 and R2, respectively.

**Example** **4.**  *Consider R1=F4+vF4 (for e=1), where $v^{2}=0$ and let $C=\langle \left(\left(1+v\right)\left(1+x+x^{2}\right);0\right),\left(0;v\left(1+x+x^{2}\right)\left(1+x^{3}+x^{6}\right)\right)\rangle$ be a double cyclic code of length (3,9) over R1. It is straightforward to verify that the code C is reversible. Since $v\left(\left(1+v\right)\left(1+x+x^{2}\right);0\right)+1\left(0;v\left(1+x+x^{2}\right)\left(1+x^{3}+x^{6}\right)\right)=\left(v+vx+vx^{2};v+vx+vx^{2}+vx^{3}+vx^{4}+vx^{5}+vx^{6}+vx^{7}+vx^{8}\right)=vI\left(x\right)\in C,$ it follows from Theorem 7 that the double cyclic code C is a reversible-complement code of length (3,9) over R1. Hence, the DNA code $C_{DNA}$ consisting of DNA strings of length 24 and having minimum distance 3, derived from $C\cup \hat{C}$, contains 124 codewords, which are listed in Table 1.*

**Example** **5.**  *For e=2, we have R2=F16+vF16 with $v^{2}=0$, and let us consider $C=\langle \left(v\left(1+x+x^{2}\right);0\right),\left(0;v\left(1+x+x^{3}\right)\left(1+x^{2}+x^{3}\right)\right)\rangle$ be a double cyclic code of length (3,7) over R2. It is easy to verify that the code C is reversible. Since $vI\left(x\right)=\left(v+vx+vx^{2};v+vx+vx^{2}+vx^{3}+vx^{4}+vx^{5}+vx^{6}\right)=1\left(v+vx+vx^{2};0\right)+1\left(0;v\left(1+x+x^{3}\right)\left(1+x^{2}+x^{3}\right)\right)\in C,$ it follows from Theorem 7 that the double cyclic code C is a reversible-complement code of length (3,7) over R2. Hence, the DNA code $C_{DNA}$ consisting of DNA strings of length *40* and having minimum distance 6, derived from $C\cup \hat{C}$, contains *496* codewords, which are presented in Table 2.*

## 4. Applications

The algebraic structures and properties developed in this work, particularly the reversible and reverse-complement double cyclic codes over the finite chain ring Re=F4e+vF4e where $v^{2}=0$, offer a wide range of practical applications. In this section, we illustrate the real-world relevance of these codes through advanced implementations, focusing on secure DNA-based data storage and authentication as well as cryptographic systems. By leveraging the guaranteed reversibility and reverse-complementarity of our codes, one can enhance data accuracy, facilitate secure key management, and achieve robust encryption in various next-generation technologies. The results established in this paper yield an explicitly measurable algebraic framework for constructing DNA-admissible linear codes with strand symmetry and efficient error detection. In particular, the generators representation $C=\langle \left(f_{0}\left(x\right)+vf_{1}\left(x\right);\;0\right),\;\left(h\left(x\right);\;g_{0}\left(x\right)+vg_{1}\left(x\right)\right)\rangle$ (Theorem 1), the divisibility relations that characterize code membership (Lemma 1 and Corollary 1), the separability criterion (Propositions 2–3), the reversibility and reverse-complement characterizations via self-reciprocal generators (Theorem 2, Proposition 5, Theorem 3), and the reverse constraint at special lengths $n_{2}=\left(2n_{3}+1\right)n_{1}$ (Theorem 4), together with the generalized Gray map from Re to the DNA alphabet, offer a systematic framework bridging ring-theoretic constructions and nucleotide sequences with verifiable guarantees.

**(A) Secure DNA data storage.** In archival storage systems, reliable decoding must account for both the uncertainty of strand orientation and the presence of biochemical noise. DNA sequences may be read in either direction or from their complementary strands, introducing forms of uncertainty that conventional codes do not tolerate. Moreover, storage and sequencing processes can introduce substitution, insertion, or deletion errors. Therefore, coding schemes designed for DNA data storage must ensure that codewords remain valid under reversal and complementation, while also providing sufficient error-correcting capability to maintain data integrity in noisy environments. The self-reciprocity conditions on $f_{0},f_{1},g_{0},g_{1}$ constitute necessary and sufficient algebraic tests for reversibility, ensuring orientation invariance by construction rather than by secondary validation steps. The Gray map preserves these invariants to quaternary strings without degrading minimum-distance calculations. For integrity checking and fast rejection of false reads, Lemma 1 yields the congruence conditions
  $$\left(f_{0}\left(x\right)+vf_{1}\left(x\right)\right)\;|\;\left(\frac{x^{n_{2}}-1}{g_{1}\left(x\right)}\;h\left(x\right)\right)\;\;\mathrm{and}\;\;\left(f_{0}\left(x\right)+vf_{1}\left(x\right)\right)\;|\;\left(v\;\frac{x^{n_{2}}-1}{g_{0}\left(x\right)}\;h\left(x\right)\right)\;\;\;\mathrm{mod}\;\left(x^{n_{1}}-1\right),$$
  which can be implemented using the same polynomial division techniques as in standard cyclic decoding. When h(x)=0, Propositions 2–3 permit independent design and decoding of the two cyclic components, which facilitates functional partitioning, such as using one block for addressing and the other for data storage. At lengths $n_{2}=\left(2n_{3}+1\right)n_{1}$, Theorem 4 provides a predictable divisibility relation between the reversed $n_{2}$-block and the $n_{1}$-block, enabling strand-invariant orientation checks and resynchronization without the need for whole-strand alignment.
**(B) DNA-based authentication.** Molecular tags and barcodes require validation methods that remain unaffected by strand orientation and can be efficiently realized using low-degree polynomial congruences. Imposing self-reciprocal generators (Theorem 2; Proposition 5; Theorem 3) guarantees that validity is invariant under reverse and reverse-complement. The derived congruences from Lemma 1 serve as local soundness conditions, rejecting any alteration that violates the underlying ring-theoretic structure even when the modification is concentrated in one coordinate block. In the case of specially constructed lengths, Theorem 4 yields an additional reversal-invariant constraint that can be verified using arithmetic in Re[x]/〈xni−1〉 where i=1,2. After applying the Gray map, these conditions serve as nucleotide-level validity checks, supporting precise false-acceptance and false-rejection analysis under sequencing error models, while remaining computationally efficient.
**(C) Cryptographic systems.** The reversible and reverse-complement DNA codes derived from double cyclic codes over Re=F4e+vF4e with $v^{2}=0$ have promising applications in cryptography. In symmetric-key cryptography, such codes enable error-resilient encryption by encoding plaintext as DNA codewords that are inherently robust against errors. A secret key can be used to select or permute these codewords, yielding ciphers that maintain data integrity and confidentiality even in noisy biological environments. For public-key cryptography, the structured codes presented here can serve as the foundation for code-based encryption schemes. In particular, one can employ a McEliece-type cryptosystem in which the public key is a generator matrix of a suitable double cyclic code over Re, while the private key leverages the known algebraic structure for efficient decoding. The large minimum distance of these codes contributes to cryptographic strength, and their efficient encoding/decoding algorithms make them attractive candidates for post-quantum code-based encryption. An important advantage of these DNA-based cryptographic constructions is their biological invariance: the codes are closed under sequence reversal and Watson-Crick complement. This means that an encrypted DNA sequence (ciphertext) remains a valid codeword even if the strand is reversed or complemented, ensuring that the decryption process still recovers the correct message. This property, along with the inherent error-correcting capabilities, enhances the robustness of DNA-based encryption and key transmission in both secure communication and authentication systems.

## 5. Conclusions

This study examined the structural properties of double cyclic codes of length $\left(n_{1},n_{2}\right)$ over the ring Re=F4e+vF4e with $v^{2}=0$, where $n_{1}$ and $n_{2}$ are odd positive integers. The notions of reversibility, complementarity, and reversible-complementarity for double cyclic codes over the ring Re were established. Utilizing these concepts, we derived necessary and sufficient conditions under which double cyclic codes of length $\left(n_{1},n_{2}\right)$ over the ring Re constituted double cyclic DNA codes, particularly when $n_{2}=\left(2n_{3}+1\right)n_{1}$ for all non-negative integers $n_{3}$. These codes demonstrated suitability for DNA code construction. Moreover, we have defined and studied a generalized Gray map for the ring Re, which converted codewords into DNA sequences over {A,T,G,C}. This mapping not only generalized known Gray maps over smaller rings $F_{2}+vF_{2}$, where $v^{2}=0$ but also preserved the algebraic properties necessary for the construction of structured DNA codes. Furthermore, we presented several illustrative examples and applications to demonstrate these codes. To demonstrate practical feasibility, the authors implemented the proposed constructions in Magma [32]. For the examples presented, code generation, application of the generalized Gray map, and verification of reverse and reverse-complement constraints were completed in a short time, indicating that the method is computationally efficient. These tasks mainly use basic ring addition and multiplication, so the time it takes increases roughly in proportion to the code length. This shows that the method works well even for larger codes. Future work may focus on analyzing the algebraic structure of double cyclic codes of various lengths and exploring additional finite rings that are suitable for DNA computing applications.

## Figures and Tables

**Table 1:** $C_{DNA}$: a DNA code of length 24 derived from $C\cup \hat{C}$ (Example 4).

AAAAAAAAAAAAAAAAAAAAAAAA	GGGGGGAAAAAAAAAAAAAAAAAA	AAAAAAAAAAAAAAAAAAGGGGGG
TTTTTTAAAAAAAAAAAAAAAAAA	AAAAAAAAAAAAAAAAAATTTTTT	CCCCCCAAAAAAAAAAAAAAAAAA
AAAAAAAAAAAAAAAAAACCCCCC	GAGAGAAAAAAAAAAAAAAAAAAA	AAAAAAAAAAAAAAAAAAGAGAGA
AGAGAGAAAAAAAAAAAAAAAAAA	AAAAAAAAAAAAAAAAAAAGAGAG	CTCTCTAAAAAAAAAAAAAAAAAA
AAAAAAAAAAAAAAAAAACTCTCT	TCTCTCAAAAAAAAAAAAAAAAAA	AAAAAAAAAAAAAAAAAATCTCTC
TATATAAAAAAAAAAAAAAAAAAA	AAAAAAAAAAAAAAAAAATATATA	CGCGCGAAAAAAAAAAAAAAAAAA
AAAAAAAAAAAAAAAAAACGCGCG	ATATATAAAAAAAAAAAAAAAAAA	AAAAAAAAAAAAAAAAAAATATAT
GCGCGCAAAAAAAAAAAAAAAAAA	AAAAAAAAAAAAAAAAAAGCGCGC	CACACAAAAAAAAAAAAAAAAAAA
AAAAAAAAAAAAAAAAAACACACA	TGTGTGAAAAAAAAAAAAAAAAAA	AAAAAAAAAAAAAAAAAATGTGTG
GTGTGTAAAAAAAAAAAAAAAAAA	AAAAAAAAAAAAAAAAAAGTGTGT	ACACACAAAAAAAAAAAAAAAAAA
AAAAAAAAAAAAAAAAAAACACAC	AAAAAAGGGGGGGGGGGGGGGGGG	GGGGGGGGGGGGGGGGGGAAAAAA
AAAAAATTTTTTTTTTTTTTTTTT	TTTTTTTTTTTTTTTTTTAAAAAA	AAAAAACCCCCCCCCCCCCCCCCC
CCCCCCCCCCCCCCCCCCAAAAAA	GGGGGGGGGGGGGGGGGGGGGGGG	GGGGGGTTTTTTTTTTTTTTTTTT
TTTTTTTTTTTTTTTTTTGGGGGG	GGGGGGCCCCCCCCCCCCCCCCCC	CCCCCCCCCCCCCCCCCCGGGGGG
TTTTTTGGGGGGGGGGGGGGGGGG	GGGGGGGGGGGGGGGGGGTTTTTT	TTTTTTTTTTTTTTTTTTTTTTTT
TTTTTTCCCCCCCCCCCCCCCCCC	CCCCCCCCCCCCCCCCCCTTTTTT	CCCCCCGGGGGGGGGGGGGGGGGG
GGGGGGGGGGGGGGGGGGCCCCCC	CCCCCCTTTTTTTTTTTTTTTTTT	TTTTTTTTTTTTTTTTTTCCCCCC
CCCCCCCCCCCCCCCCCCCCCCCC	GAGAGAGGGGGGGGGGGGGGGGGG	GGGGGGGGGGGGGGGGGGGAGAGA
GAGAGATTTTTTTTTTTTTTTTTT	TTTTTTTTTTTTTTTTTTGAGAGA	GAGAGACCCCCCCCCCCCCCCCCC
CCCCCCCCCCCCCCCCCCGAGAGA	AGAGAGGGGGGGGGGGGGGGGGGG	GGGGGGGGGGGGGGGGGGAGAGAG
AGAGAGTTTTTTTTTTTTTTTTTT	TTTTTTTTTTTTTTTTTTAGAGAG	AGAGAGCCCCCCCCCCCCCCCCCC
CCCCCCCCCCCCCCCCCCAGAGAG	CTCTCTGGGGGGGGGGGGGGGGGG	GGGGGGGGGGGGGGGGGGCTCTCT
CTCTCTTTTTTTTTTTTTTTTTTT	TTTTTTTTTTTTTTTTTTCTCTCT	CTCTCTCCCCCCCCCCCCCCCCCC
CCCCCCCCCCCCCCCCCCCTCTCT	TCTCTCGGGGGGGGGGGGGGGGGG	GGGGGGGGGGGGGGGGGGTCTCTC
TCTCTCTTTTTTTTTTTTTTTTTT	TTTTTTTTTTTTTTTTTTTCTCTC	TCTCTCCCCCCCCCCCCCCCCCCC
CCCCCCCCCCCCCCCCCTCTCTC	TATATAGGGGGGGGGGGGGGGGGG	GGGGGGGGGGGGGGGGGGTATATA
TATATATTTTTTTTTTTTTTTTTT	TTTTTTTTTTTTTTTTTTATATA	TATATACCCCCCCCCCCCCCCCCC
CCCCCCCCCCCCCCCCCTATATA	CGCGCGGGGGGGGGGGGGGGGGGG	GGGGGGGGGGGGGGGGGGCGCGCG
CGCGCGTTTTTTTTTTTTTTTTTT	TTTTTTTTTTTTTTTTTTCGCGCG	CGCGCGCCCCCCCCCCCCCCCCCC
CCCCCCCCCCCCCCCCCCCGCGCG	ATATATGGGGGGGGGGGGGGGGGG	GGGGGGGGGGGGGGGGGGATATAT
ATATATTTTTTTTTTTTTTTTTTT	TTTTTTTTTTTTTTTTTTATATAT	ATATATCCCCCCCCCCCCCCCCCC
CCCCCCCCCCCCCCCCCCATATAT	GCGCGCGGGGGGGGGGGGGGGGGG	GGGGGGGGGGGGGGGGGGCGCGC
GCGCGCTTTTTTTTTTTTTTTTTT	TTTTTTTTTTTTTTTTTTGCGCGC	GCGCGCCCCCCCCCCCCCCCCCCC
CCCCCCCCCCCCCCCCCCGCGCGC	CACACAGGGGGGGGGGGGGGGGGG	GGGGGGGGGGGGGGGGGGCACACA
CACACATTTTTTTTTTTTTTTTTT	TTTTTTTTTTTTTTTTTTCACACA	CACACACCCCCCCCCCCCCCCCCC
CCCCCCCCCCCCCCCCCCCACACA	TGTGTGGGGGGGGGGGGGGGGGGG	GGGGGGGGGGGGGGGGGGGTGTGTG
TGTGTGTTTTTTTTTTTTTTTTTT	TTTTTTTTTTTTTTTTTTTGTGTG	TGTGTGCCCCCCCCCCCCCCCCCC
CCCCCCCCCCCCCCCCCTGTGTG	GTGTGTGGGGGGGGGGGGGGGGGG	GGGGGGGGGGGGGGGGGGGTGTGT
GTGTGTTTTTTTTTTTTTTTTTTT	TTTTTTTTTTTTTTTTTTGTGTGT	GTGTGTCCCCCCCCCCCCCCCCCC
CCCCCCCCCCCCCCCCCCGTGTGT	ACACACGGGGGGGGGGGGGGGGGG	GGGGGGGGGGGGGGGGGGACACAC
ACACACTTTTTTTTTTTTTTTTTT	TTTTTTTTTTTTTTTTTTACACAC	ACACACCCCCCCCCCCCCCCCCCC
CCCCCCCCCCCCCCCCCCACACAC		

**Table 2:** $C_{DNA}$: a DNA code of length 40 derived from $C\cup \hat{C}$ (Example 5).

AAAAAAAAAAAAAAAAAAAAAAAAAAAAAAAAAAAAAAAA	GAGAGAGAGAGAAAAAAAAAAAAAAAAAAAAAAAAAAAAA
AAAAAAAAAAAAAAAAAAAAAAAAAAAAGAGAGAGAGAGA	AGAGAGAGAGAGAAAAAAAAAAAAAAAAAAAAAAAAAAAA
AAAAAAAAAAAAAAAAAAAAAAAAAAAAAGAGAGAGAGAG	TGTGTGTGTGTGAAAAAAAAAAAAAAAAAAAAAAAAAAAA
AAAAAAAAAAAAAAAAAAAAAAAAAAAATGTGTGTGTGTG	TCTCTCTCTCTCAAAAAAAAAAAAAAAAAAAAAAAAAAAA
AAAAAAAAAAAAAAAAAAAAAAAAAAAATCTCTCTCTCTC	GGGGGGGGGGGGAAAAAAAAAAAAAAAAAAAAAAAAAAAA
AAAAAAAAAAAAAAAAAAAAAAAAAAAAGGGGGGGGGGGG	TATATATATATAAAAAAAAAAAAAAAAAAAAAAAAAAAAA
AAAAAAAAAAAAAAAAAAAAAAAAAAAATATATATATATA	ATATATATATATAAAAAAAAAAAAAAAAAAAAAAAAAAAA
AAAAAAAAAAAAAAAAAAAAAAAAAAAAATATATATATAT	CTCTCTCTCTCTAAAAAAAAAAAAAAAAAAAAAAAAAAAA
AAAAAAAAAAAAAAAAAAAAAAAAAAAACTCTCTCTCTCT	CGCGCGCGCGCGAAAAAAAAAAAAAAAAAAAAAAAAAAAA
AAAAAAAAAAAAAAAAAAAAAAAAAAAACGCGCGCGCGCG	TTTTTTTTTTTTAAAAAAAAAAAAAAAAAAAAAAAAAAAA
AAAAAAAAAAAAAAAAAAAAAAAAAAAATTTTTTTTTTTT	CACACACACACAAAAAAAAAAAAAAAAAAAAAAAAAAAAA
AAAAAAAAAAAAAAAAAAAAAAAAAAAACACACACACACA	ACACACACACACAAAAAAAAAAAAAAAAAAAAAAAAAAAA
AAAAAAAAAAAAAAAAAAAAAAAAAAAAACACACACACAC	GCGCGCGCGCGCAAAAAAAAAAAAAAAAAAAAAAAAAAAA
AAAAAAAAAAAAAAAAAAAAAAAAAAAAGCGCGCGCGCGC	GTGTGTGTGTGTAAAAAAAAAAAAAAAAAAAAAAAAAAAA
AAAAAAAAAAAAAAAAAAAAAAAAAAAAGTGTGTGTGTGT	CCCCCCCCCCCCAAAAAAAAAAAAAAAAAAAAAAAAAAAA
AAAAAAAAAAAAAAAAAAAAAAAAAAAACCCCCCCCCCCC	AAAAAAAAAAAAGAGAGAGAGAGAGAGAGAGAGAGAGAGA
GAGAGAGAGAGAGAGAGAGAGAGAGAGAAAAAAAAAAAAA	AAAAAAAAAAAAAGAGAGAGAGAGAGAGAGAGAGAGAGAG
AGAGAGAGAGAGAGAGAGAGAGAGAGAGAAAAAAAAAAAA	AAAAAAAAAAAATGTGTGTGTGTGTGTGTGTGTGTGTGTG
TGTGTGTGTGTGTGTGTGTGTGTGTGTGAAAAAAAAAAAA	AAAAAAAAAAAATCTCTCTCTCTCTCTCTCTCTCTCTCTC
TCTCTCTCTCTCTCTCTCTCTCTCTCTCAAAAAAAAAAAA	AAAAAAAAAAAAGGGGGGGGGGGGGGGGGGGGGGGGGGGG
GGGGGGGGGGGGGGGGGGGGGGGGGGGGAAAAAAAAAAAA	AAAAAAAAAAAATATATATATATATATATATATATATATA
TATATATATATATATATATATATATATAAAAAAAAAAAAA	AAAAAAAAAAAAATATATATATATATATATATATATATAT
ATATATATATATATATATATATATATATAAAAAAAAAAAA	AAAAAAAAAAAACTCTCTCTCTCTCTCTCTCTCTCTCTCT
CTCTCTCTCTCTCTCTCTCTCTCTCTCTAAAAAAAAAAAA	AAAAAAAAAAAACGCGCGCGCGCGCGCGCGCGCGCGCGCG
CGCGCGCGCGCGCGCGCGCGCGCGCGCGAAAAAAAAAAAA	AAAAAAAAAAAATTTTTTTTTTTTTTTTTTTTTTTTTTTT
TTTTTTTTTTTTTTTTTTTTTTTTTTTTAAAAAAAAAAAA	AAAAAAAAAAAACACACACACACACACACACACACACACA
CACACACACACACACACACACACACACAAAAAAAAAAAAA	AAAAAAAAAAAAACACACACACACACACACACACACACAC
ACACACACACACACACACACACACACACAAAAAAAAAAAA	AAAAAAAAAAAAGCGCGCGCGCGCGCGCGCGCGCGCGCGC
GCGCGCGCGCGCGCGCGCGCGCGCGCGCAAAAAAAAAAAA	AAAAAAAAAAAAGTGTGTGTGTGTGTGTGTGTGTGTGTGT
GTGTGTGTGTGTGTGTGTGTGTGTGTGTAAAAAAAAAAAA	AAAAAAAAAAAACCCCCCCCCCCCCCCCCCCCCCCCCCCC
CCCCCCCCCCCCCCCCCCCCCCCCCCCCAAAAAAAAAAAA	GAGAGAGAGAGAGAGAGAGAGAGAGAGAGAGAGAGAGAGA
GAGAGAGAGAGAAGAGAGAGAGAGAGAGAGAGAGAGAGAG	AGAGAGAGAGAGAGAGAGAGAGAGAGAGGAGAGAGAGAGA
GAGAGAGAGAGATGTGTGTGTGTGTGTGTGTGTGTGTGTG	TGTGTGTGTGTGTGTGTGTGTGTGTGTGGAGAGAGAGAGA
GAGAGAGAGAGATCTCTCTCTCTCTCTCTCTCTCTCTCTC	TCTCTCTCTCTCTCTCTCTCTCTCTCTCGAGAGAGAGAGA
GAGAGAGAGAGAGGGGGGGGGGGGGGGGGGGGGGGGGGGG	GGGGGGGGGGGGGGGGGGGGGGGGGGGGGAGAGAGAGAGA
GAGAGAGAGAGATATATATATATATATATATATATATATA	TATATATATATATATATATATATATATAGAGAGAGAGAGA
GAGAGAGAGAGAATATATATATATATATATATATATATAT	ATATATATATATATATATATATATATATGAGAGAGAGAGA
GAGAGAGAGAGACTCTCTCTCTCTCTCTCTCTCTCTCTCT	CTCTCTCTCTCTCTCTCTCTCTCTCTCTGAGAGAGAGAGA
GAGAGAGAGAGACGCGCGCGCGCGCGCGCGCGCGCGCGCG	CGCGCGCGCGCGCGCGCGCGCGCGCGCGGAGAGAGAGAGA
GAGAGAGAGAGATTTTTTTTTTTTTTTTTTTTTTTTTTTT	TTTTTTTTTTTTTTTTTTTTTTTTTTTTGAGAGAGAGAGA
GAGAGAGAGAGACACACACACACACACACACACACACACA	CACACACACACACACACACACACACACAGAGAGAGAGAGA
GAGAGAGAGAGAACACACACACACACACACACACACACAC	ACACACACACACACACACACACACACACGAGAGAGAGAGA
GAGAGAGAGAGAGCGCGCGCGCGCGCGCGCGCGCGCGCGC	GCGCGCGCGCGCGCGCGCGCGCGCGCGCGAGAGAGAGAGA
GAGAGAGAGAGAGTGTGTGTGTGTGTGTGTGTGTGTGTGT	GTGTGTGTGTGTGTGTGTGTGTGTGTGTGAGAGAGAGAGA
GAGAGAGAGAGACCCCCCCCCCCCCCCCCCCCCCCCCCCC	CCCCCCCCCCCCCCCCCCCCCCCCCCCCGAGAGAGAGAGA
AGAGAGAGAGAGGAGAGAGAGAGAGAGAGAGAGAGAGAGA	GAGAGAGAGAGAGAGAGAGAGAGAGAGAAGAGAGAGAGAG
AGAGAGAGAGAGAGAGAGAGAGAGAGAGAGAGAGAGAGAG	AGAGAGAGAGAGTGTGTGTGTGTGTGTGTGTGTGTGTGTG
TGTGTGTGTGTGTGTGTGTGTGTGTGTGAGAGAGAGAGAG	AGAGAGAGAGAGTCTCTCTCTCTCTCTCTCTCTCTCTCTC
TCTCTCTCTCTCTCTCTCTCTCTCTCTCAGAGAGAGAGAG	AGAGAGAGAGAGGGGGGGGGGGGGGGGGGGGGGGGGGGGG
GGGGGGGGGGGGGGGGGGGGGGGGGGGGAGAGAGAGAGAG	AGAGAGAGAGAGTATATATATATATATATATATATATATA
TATATATATATATATATATATATATATAAGAGAGAGAGAG	AGAGAGAGAGAGATATATATATATATATATATATATATAT
ATATATATATATATATATATATATATATAGAGAGAGAGAG	AGAGAGAGAGAGCTCTCTCTCTCTCTCTCTCTCTCTCTCT
CTCTCTCTCTCTCTCTCTCTCTCTCTCTAGAGAGAGAGAG	AGAGAGAGAGAGCGCGCGCGCGCGCGCGCGCGCGCGCGCG
CGCGCGCGCGCGCGCGCGCGCGCGCGCGAGAGAGAGAGAG	AGAGAGAGAGAGTTTTTTTTTTTTTTTTTTTTTTTTTTTT
TTTTTTTTTTTTTTTTTTTTTTTTTTTTAGAGAGAGAGAG	AGAGAGAGAGAGCACACACACACACACACACACACACACA
CACACACACACACACACACACACACACAAGAGAGAGAGAG	AGAGAGAGAGAGACACACACACACACACACACACACACAC
ACACACACACACACACACACACACACACAGAGAGAGAGAG	AGAGAGAGAGAGGCGCGCGCGCGCGCGCGCGCGCGCGCGC
GCGCGCGCGCGCGCGCGCGCGCGCGCGCAGAGAGAGAGAG	AGAGAGAGAGAGGTGTGTGTGTGTGTGTGTGTGTGTGTGT
GTGTGTGTGTGTGTGTGTGTGTGTGTGTAGAGAGAGAGAG	AGAGAGAGAGAGCCCCCCCCCCCCCCCCCCCCCCCCCCCC
CCCCCCCCCCCCCCCCCCCCCCCCCCCCAGAGAGAGAGAG	TGTGTGTGTGTGGAGAGAGAGAGAGAGAGAGAGAGAGAGA
GAGAGAGAGAGAGAGAGAGAGAGAGAGATGTGTGTGTGTG	TGTGTGTGTGTGAGAGAGAGAGAGAGAGAGAGAGAGAGAG
AGAGAGAGAGAGAGAGAGAGAGAGAGAGTGTGTGTGTGTG	TGTGTGTGTGTGTGTGTGTGTGTGTGTGTGTGTGTGTGTG
TGTGTGTGTGTGTCTCTCTCTCTCTCTCTCTCTCTCTCTC	TCTCTCTCTCTCTCTCTCTCTCTCTCTCTGTGTGTGTGTG
TGTGTGTGTGTGGGGGGGGGGGGGGGGGGGGGGGGGGGGG	GGGGGGGGGGGGGGGGGGGGGGGGGGGGTGTGTGTGTGTG
TGTGTGTGTGTGTATATATATATATATATATATATATATA	TATATATATATATATATATATATATATATGTGTGTGTGTG
TGTGTGTGTGTGATATATATATATATATATATATATATAT	ATATATATATATATATATATATATATATTGTGTGTGTGTG
TGTGTGTGTGTGCTCTCTCTCTCTCTCTCTCTCTCTCTCT	CTCTCTCTCTCTCTCTCTCTCTCTCTCTTGTGTGTGTGTG
TGTGTGTGTGTGCGCGCGCGCGCGCGCGCGCGCGCGCGCG	CGCGCGCGCGCGCGCGCGCGCGCGCGCGTGTGTGTGTGTG
TGTGTGTGTGTGTTTTTTTTTTTTTTTTTTTTTTTTTTTT	TTTTTTTTTTTTTTTTTTTTTTTTTTTTTGTGTGTGTGTG
TGTGTGTGTGTGCACACACACACACACACACACACACACA	CACACACACACACACACACACACACACATGTGTGTGTGTG
TGTGTGTGTGTGACACACACACACACACACACACACACAC	ACACACACACACACACACACACACACACTGTGTGTGTGTG
TGTGTGTGTGTGGCGCGCGCGCGCGCGCGCGCGCGCGCGC	GCGCGCGCGCGCGCGCGCGCGCGCGCGCTGTGTGTGTGTG
TGTGTGTGTGTGGTGTGTGTGTGTGTGTGTGTGTGTGTGT	GTGTGTGTGTGTGTGTGTGTGTGTGTGTTGTGTGTGTGTG
TGTGTGTGTGTGCCCCCCCCCCCCCCCCCCCCCCCCCCCC	CCCCCCCCCCCCCCCCCCCCCCCCCCCCTGTGTGTGTGTG
TCTCTCTCTCTCGAGAGAGAGAGAGAGAGAGAGAGAGAGA	GAGAGAGAGAGAGAGAGAGAGAGAGAGATCTCTCTCTCTC
TCTCTCTCTCTCAGAGAGAGAGAGAGAGAGAGAGAGAGAG	AGAGAGAGAGAGAGAGAGAGAGAGAGAGTCTCTCTCTCTC
TCTCTCTCTCTCTGTGTGTGTGTGTGTGTGTGTGTGTGTG	TGTGTGTGTGTGTGTGTGTGTGTGTGTGTCTCTCTCTCTC
TCTCTCTCTCTCTCTCTCTCTCTCTCTCTCTCTCTCTCTC	TCTCTCTCTCTCGGGGGGGGGGGGGGGGGGGGGGGGGGGG
GGGGGGGGGGGGGGGGGGGGGGGGGGGGTCTCTCTCTCTC	TCTCTCTCTCTCTATATATATATATATATATATATATATA
TATATATATATATATATATATATATATATCTCTCTCTCTC	TCTCTCTCTCTCATATATATATATATATATATATATATAT
ATATATATATATATATATATATATATATTCTCTCTCTCTC	TCTCTCTCTCTCCTCTCTCTCTCTCTCTCTCTCTCTCTCT
CTCTCTCTCTCTCTCTCTCTCTCTCTCTTCTCTCTCTCTC	TCTCTCTCTCTCCGCGCGCGCGCGCGCGCGCGCGCGCGCG
CGCGCGCGCGCGCGCGCGCGCGCGCGCGTCTCTCTCTCTC	TCTCTCTCTCTCTTTTTTTTTTTTTTTTTTTTTTTTTTTT
TTTTTTTTTTTTTTTTTTTTTTTTTTTTTCTCTCTCTCTC	TCTCTCTCTCTCCACACACACACACACACACACACACACA
CACACACACACACACACACACACACACATCTCTCTCTCTC	TCTCTCTCTCTCACACACACACACACACACACACACACAC
ACACACACACACACACACACACACACACTCTCTCTCTCTC	TCTCTCTCTCTCGCGCGCGCGCGCGCGCGCGCGCGCGCGC
GCGCGCGCGCGCGCGCGCGCGCGCGCGCTCTCTCTCTCTC	TCTCTCTCTCTCGTGTGTGTGTGTGTGTGTGTGTGTGTGT
GTGTGTGTGTGTGTGTGTGTGTGTGTGTTCTCTCTCTCTC	TCTCTCTCTCTCCCCCCCCCCCCCCCCCCCCCCCCCCCCC
CCCCCCCCCCCCCCCCCCCCCCCCCCCCTCTCTCTCTCTC	GGGGGGGGGGGGGAGAGAGAGAGAGAGAGAGAGAGAGAGA
GAGAGAGAGAGAGAGAGAGAGAGAGAGAGGGGGGGGGGGG	GGGGGGGGGGGGAGAGAGAGAGAGAGAGAGAGAGAGAGAG
AGAGAGAGAGAGAGAGAGAGAGAGAGAGGGGGGGGGGGGG	GGGGGGGGGGGGTGTGTGTGTGTGTGTGTGTGTGTGTGTG
TGTGTGTGTGTGTGTGTGTGTGTGTGTGGGGGGGGGGGGG	GGGGGGGGGGGGTCTCTCTCTCTCTCTCTCTCTCTCTCTC
TCTCTCTCTCTCTCTCTCTCTCTCTCTCGGGGGGGGGGGG	GGGGGGGGGGGGGGGGGGGGGGGGGGGGGGGGGGGGGGGG
GGGGGGGGGGGGTATATATATATATATATATATATATATA	TATATATATATATATATATATATATATAGGGGGGGGGGGG
GGGGGGGGGGGGATATATATATATATATATATATATATAT	ATATATATATATATATATATATATATATGGGGGGGGGGGG
GGGGGGGGGGGGCTCTCTCTCTCTCTCTCTCTCTCTCTCT	CTCTCTCTCTCTCTCTCTCTCTCTCTCTGGGGGGGGGGGG
GGGGGGGGGGGGCGCGCGCGCGCGCGCGCGCGCGCGCGCG	CGCGCGCGCGCGCGCGCGCGCGCGCGCGGGGGGGGGGGGG
GGGGGGGGGGGGTTTTTTTTTTTTTTTTTTTTTTTTTTTT	TTTTTTTTTTTTTTTTTTTTTTTTTTTTGGGGGGGGGGGG
GGGGGGGGGGGGCACACACACACACACACACACACACACA	CACACACACACACACACACACACACACAGGGGGGGGGGGG
GGGGGGGGGGGGACACACACACACACACACACACACACAC	ACACACACACACACACACACACACACACGGGGGGGGGGGG
GGGGGGGGGGGGGCGCGCGCGCGCGCGCGCGCGCGCGCGC	GCGCGCGCGCGCGCGCGCGCGCGCGCGCGGGGGGGGGGGG
GGGGGGGGGGGGGTGTGTGTGTGTGTGTGTGTGTGTGTGT	GTGTGTGTGTGTGTGTGTGTGTGTGTGTGGGGGGGGGGGG
GGGGGGGGGGGGCCCCCCCCCCCCCCCCCCCCCCCCCCCC	CCCCCCCCCCCCCCCCCCCCCCCCCCCCGGGGGGGGGGGG
TATATATATATAGAGAGAGAGAGAGAGAGAGAGAGAGAGA	GAGAGAGAGAGAGAGAGAGAGAGAGAGATATATATATATA
TATATATATATAAGAGAGAGAGAGAGAGAGAGAGAGAGAG	AGAGAGAGAGAGAGAGAGAGAGAGAGAGTATATATATATA
TATATATATATATGTGTGTGTGTGTGTGTGTGTGTGTGTG	TGTGTGTGTGTGTGTGTGTGTGTGTGTGTATATATATATA
TATATATATATATCTCTCTCTCTCTCTCTCTCTCTCTCTC	TCTCTCTCTCTCTCTCTCTCTCTCTCTCTATATATATATA
TATATATATATAGGGGGGGGGGGGGGGGGGGGGGGGGGGG	GGGGGGGGGGGGGGGGGGGGGGGGGGGGTATATATATATA
TATATATATATATATATATATATATATATATATATATATA	TATATATATATAATATATATATATATATATATATATATAT
ATATATATATATATATATATATATATATTATATATATATA	TATATATATATACTCTCTCTCTCTCTCTCTCTCTCTCTCT
CTCTCTCTCTCTCTCTCTCTCTCTCTCTTATATATATATA	TATATATATATACGCGCGCGCGCGCGCGCGCGCGCGCGCG
CGCGCGCGCGCGCGCGCGCGCGCGCGCGTATATATATATA	TATATATATATATTTTTTTTTTTTTTTTTTTTTTTTTTTT
TTTTTTTTTTTTTTTTTTTTTTTTTTTTTATATATATATA	TATATATATATACACACACACACACACACACACACACACA
CACACACACACACACACACACACACACATATATATATATA	TATATATATATAACACACACACACACACACACACACACAC
ACACACACACACACACACACACACACACTATATATATATA	TATATATATATAGCGCGCGCGCGCGCGCGCGCGCGCGCGC
GCGCGCGCGCGCGCGCGCGCGCGCGCGCTATATATATATA	TATATATATATAGTGTGTGTGTGTGTGTGTGTGTGTGTGT
GTGTGTGTGTGTGTGTGTGTGTGTGTGTTATATATATATA	TATATATATATACCCCCCCCCCCCCCCCCCCCCCCCCCCC
CCCCCCCCCCCCCCCCCCCCCCCCCCCCTATATATATATA	ATATATATATATGAGAGAGAGAGAGAGAGAGAGAGAGAGA
GAGAGAGAGAGAGAGAGAGAGAGAGAGAATATATATATAT	ATATATATATATAGAGAGAGAGAGAGAGAGAGAGAGAGAG
AGAGAGAGAGAGAGAGAGAGAGAGAGAGATATATATATAT	ATATATATATATTGTGTGTGTGTGTGTGTGTGTGTGTGTG
TGTGTGTGTGTGTGTGTGTGTGTGTGTGATATATATATAT	ATATATATATATTCTCTCTCTCTCTCTCTCTCTCTCTCTC
TCTCTCTCTCTCTCTCTCTCTCTCTCTCATATATATATAT	ATATATATATATGGGGGGGGGGGGGGGGGGGGGGGGGGGG
GGGGGGGGGGGGGGGGGGGGGGGGGGGGATATATATATAT	ATATATATATATTATATATATATATATATATATATATATA
TATATATATATATATATATATATATATAATATATATATAT	ATATATATATATATATATATATATATATATATATATATAT
ATATATATATATCTCTCTCTCTCTCTCTCTCTCTCTCTCT	CTCTCTCTCTCTCTCTCTCTCTCTCTCTATATATATATAT
ATATATATATATCGCGCGCGCGCGCGCGCGCGCGCGCGCG	CGCGCGCGCGCGCGCGCGCGCGCGCGCGATATATATATAT
ATATATATATATTTTTTTTTTTTTTTTTTTTTTTTTTTTT	TTTTTTTTTTTTTTTTTTTTTTTTTTTTATATATATATAT
ATATATATATATCACACACACACACACACACACACACACA	CACACACACACACACACACACACACACAATATATATATAT
ATATATATATATACACACACACACACACACACACACACAC	ACACACACACACACACACACACACACACATATATATATAT
ATATATATATATGCGCGCGCGCGCGCGCGCGCGCGCGCGC	GCGCGCGCGCGCGCGCGCGCGCGCGCGCATATATATATAT
ATATATATATATGTGTGTGTGTGTGTGTGTGTGTGTGTGT	GTGTGTGTGTGTGTGTGTGTGTGTGTGTATATATATATAT
ATATATATATATCCCCCCCCCCCCCCCCCCCCCCCCCCCC	CCCCCCCCCCCCCCCCCCCCCCCCCCCCATATATATATAT
CTCTCTCTCTCTGAGAGAGAGAGAGAGAGAGAGAGAGAGA	GAGAGAGAGAGAGAGAGAGAGAGAGAGACTCTCTCTCTCT
CTCTCTCTCTCTAGAGAGAGAGAGAGAGAGAGAGAGAGAG	AGAGAGAGAGAGAGAGAGAGAGAGAGAGCTCTCTCTCTCT
CTCTCTCTCTCTTGTGTGTGTGTGTGTGTGTGTGTGTGTG	TGTGTGTGTGTGTGTGTGTGTGTGTGTGCTCTCTCTCTCT
CTCTCTCTCTCTTCTCTCTCTCTCTCTCTCTCTCTCTCTC	TCTCTCTCTCTCTCTCTCTCTCTCTCTCCTCTCTCTCTCT
CTCTCTCTCTCTGGGGGGGGGGGGGGGGGGGGGGGGGGGG	GGGGGGGGGGGGGGGGGGGGGGGGGGGGCTCTCTCTCTCT
CTCTCTCTCTCTTATATATATATATATATATATATATATA	TATATATATATATATATATATATATATACTCTCTCTCTCT
CTCTCTCTCTCTATATATATATATATATATATATATATAT	ATATATATATATATATATATATATATATCTCTCTCTCTCT
CTCTCTCTCTCTCTCTCTCTCTCTCTCTCTCTCTCTCTCT	CTCTCTCTCTCTCGCGCGCGCGCGCGCGCGCGCGCGCGCG
CGCGCGCGCGCGCGCGCGCGCGCGCGCGCTCTCTCTCTCT	CTCTCTCTCTCTTTTTTTTTTTTTTTTTTTTTTTTTTTTT
TTTTTTTTTTTTTTTTTTTTTTTTTTTTCTCTCTCTCTCT	CTCTCTCTCTCTCACACACACACACACACACACACACACA
CACACACACACACACACACACACACACACTCTCTCTCTCT	CTCTCTCTCTCTACACACACACACACACACACACACACAC
ACACACACACACACACACACACACACACCTCTCTCTCTCT	CTCTCTCTCTCTGCGCGCGCGCGCGCGCGCGCGCGCGCGC
GCGCGCGCGCGCGCGCGCGCGCGCGCGCCTCTCTCTCTCT	CTCTCTCTCTCTGTGTGTGTGTGTGTGTGTGTGTGTGTGT
GTGTGTGTGTGTGTGTGTGTGTGTGTGTCTCTCTCTCTCT	CTCTCTCTCTCTCCCCCCCCCCCCCCCCCCCCCCCCCCCC
CCCCCCCCCCCCCCCCCCCCCCCCCCCCCTCTCTCTCTCT	CGCGCGCGCGCGGAGAGAGAGAGAGAGAGAGAGAGAGAGA
GAGAGAGAGAGAGAGAGAGAGAGAGAGACGCGCGCGCGCG	CGCGCGCGCGCGAGAGAGAGAGAGAGAGAGAGAGAGAGAG
AGAGAGAGAGAGAGAGAGAGAGAGAGAGCGCGCGCGCGCG	CGCGCGCGCGCGTGTGTGTGTGTGTGTGTGTGTGTGTGTG
TGTGTGTGTGTGTGTGTGTGTGTGTGTGCGCGCGCGCGCG	CGCGCGCGCGCGTCTCTCTCTCTCTCTCTCTCTCTCTCTC
TCTCTCTCTCTCTCTCTCTCTCTCTCTCCGCGCGCGCGCG	CGCGCGCGCGCGGGGGGGGGGGGGGGGGGGGGGGGGGGGG
GGGGGGGGGGGGGGGGGGGGGGGGGGGGGCGCGCGCGCGC	CGCGCGCGCGCGTATATATATATATATATATATATATATA
TATATATATATATATATATATATATATACGCGCGCGCGCG	CGCGCGCGCGCGATATATATATATATATATATATATATAT
ATATATATATATATATATATATATATATCGCGCGCGCGCG	CGCGCGCGCGCGCTCTCTCTCTCTCTCTCTCTCTCTCTCT
CTCTCTCTCTCTCTCTCTCTCTCTCTCTCGCGCGCGCGCG	CGCGCGCGCGCGCGCGCGCGCGCGCGCGCGCGCGCGCGCG
CGCGCGCGCGCGTTTTTTTTTTTTTTTTTTTTTTTTTTTT	TTTTTTTTTTTTTTTTTTTTTTTTTTTTCGCGCGCGCGCG
CGCGCGCGCGCGCACACACACACACACACACACACACACA	CACACACACACACACACACACACACACACGCGCGCGCGCG
CGCGCGCGCGCGACACACACACACACACACACACACACAC	ACACACACACACACACACACACACACACCGCGCGCGCGCG
CGCGCGCGCGCGGCGCGCGCGCGCGCGCGCGCGCGCGCGC	GCGCGCGCGCGCGCGCGCGCGCGCGCGCCGCGCGCGCGCG
CGCGCGCGCGCGGTGTGTGTGTGTGTGTGTGTGTGTGTGT	GTGTGTGTGTGTGTGTGTGTGTGTGTGTCGCGCGCGCGCG
CGCGCGCGCGCGCCCCCCCCCCCCCCCCCCCCCCCCCCCC	CCCCCCCCCCCCCCCCCCCCCCCCCCCCCGCGCGCGCGCG
TTTTTTTTTTTTGAGAGAGAGAGAGAGAGAGAGAGAGAGA	GAGAGAGAGAGAGAGAGAGAGAGAGAGATTTTTTTTTTTT
TTTTTTTTTTTTAGAGAGAGAGAGAGAGAGAGAGAGAGAG	AGAGAGAGAGAGAGAGAGAGAGAGAGAGTTTTTTTTTTTT
TTTTTTTTTTTTTGTGTGTGTGTGTGTGTGTGTGTGTGTG	TGTGTGTGTGTGTGTGTGTGTGTGTGTGTTTTTTTTTTTT
TTTTTTTTTTTTTCTCTCTCTCTCTCTCTCTCTCTCTCTC	TCTCTCTCTCTCTCTCTCTCTCTCTCTCTTTTTTTTTTTT
TTTTTTTTTTTTGGGGGGGGGGGGGGGGGGGGGGGGGGGG	GGGGGGGGGGGGGGGGGGGGGGGGGGGGTTTTTTTTTTTT
TTTTTTTTTTTTTATATATATATATATATATATATATATA	TATATATATATATATATATATATATATATTTTTTTTTTTT
TTTTTTTTTTTTATATATATATATATATATATATATATAT	ATATATATATATATATATATATATATATTTTTTTTTTTTT
TTTTTTTTTTTTCTCTCTCTCTCTCTCTCTCTCTCTCTCT	CTCTCTCTCTCTCTCTCTCTCTCTCTCTTTTTTTTTTTTT
TTTTTTTTTTTTCGCGCGCGCGCGCGCGCGCGCGCGCGCG	CGCGCGCGCGCGCGCGCGCGCGCGCGCGTTTTTTTTTTTT
TTTTTTTTTTTTTTTTTTTTTTTTTTTTTTTTTTTTTTTT	TTTTTTTTTTTTCACACACACACACACACACACACACACA
CACACACACACACACACACACACACACATTTTTTTTTTTT	TTTTTTTTTTTTACACACACACACACACACACACACACAC
ACACACACACACACACACACACACACACTTTTTTTTTTTT	TTTTTTTTTTTTGCGCGCGCGCGCGCGCGCGCGCGCGCGC
GCGCGCGCGCGCGCGCGCGCGCGCGCGCTTTTTTTTTTTT	TTTTTTTTTTTTGTGTGTGTGTGTGTGTGTGTGTGTGTGT
GTGTGTGTGTGTGTGTGTGTGTGTGTGTTTTTTTTTTTTT	TTTTTTTTTTTTCCCCCCCCCCCCCCCCCCCCCCCCCCCC
CCCCCCCCCCCCCCCCCCCCCCCCCCCCTTTTTTTTTTTT	CACACACACACAGAGAGAGAGAGAGAGAGAGAGAGAGAGA
GAGAGAGAGAGAGAGAGAGAGAGAGAGACACACACACACA	CACACACACACAAGAGAGAGAGAGAGAGAGAGAGAGAGAG
AGAGAGAGAGAGAGAGAGAGAGAGAGAGCACACACACACA	CACACACACACATGTGTGTGTGTGTGTGTGTGTGTGTGTG
TGTGTGTGTGTGTGTGTGTGTGTGTGTGCACACACACACA	CACACACACACATCTCTCTCTCTCTCTCTCTCTCTCTCTC
TCTCTCTCTCTCTCTCTCTCTCTCTCTCCACACACACACA	CACACACACACAGGGGGGGGGGGGGGGGGGGGGGGGGGGG
GGGGGGGGGGGGGGGGGGGGGGGGGGGGCACACACACACA	CACACACACACATATATATATATATATATATATATATATA
TATATATATATATATATATATATATATACACACACACACA	CACACACACACAATATATATATATATATATATATATATAT
ATATATATATATATATATATATATATATCACACACACACA	CACACACACACACTCTCTCTCTCTCTCTCTCTCTCTCTCT
CTCTCTCTCTCTCTCTCTCTCTCTCTCTCACACACACACA	CACACACACACACGCGCGCGCGCGCGCGCGCGCGCGCGCG
CGCGCGCGCGCGCGCGCGCGCGCGCGCGCACACACACACA	CACACACACACATTTTTTTTTTTTTTTTTTTTTTTTTTTT
TTTTTTTTTTTTTTTTTTTTTTTTTTTTCACACACACACA	CACACACACACACACACACACACACACACACACACACACA
CACACACACACAACACACACACACACACACACACACACAC	ACACACACACACACACACACACACACACCACACACACACA
CACACACACACAGCGCGCGCGCGCGCGCGCGCGCGCGCGC	GCGCGCGCGCGCGCGCGCGCGCGCGCGCCACACACACACA
CACACACACACAGTGTGTGTGTGTGTGTGTGTGTGTGTGT	GTGTGTGTGTGTGTGTGTGTGTGTGTGTCACACACACACA
CACACACACACACCCCCCCCCCCCCCCCCCCCCCCCCCCC	CCCCCCCCCCCCCCCCCCCCCCCCCCCCCACACACACACA
ACACACACACACGAGAGAGAGAGAGAGAGAGAGAGAGAGA	GAGAGAGAGAGAGAGAGAGAGAGAGAGAACACACACACAC
ACACACACACACAGAGAGAGAGAGAGAGAGAGAGAGAGAG	AGAGAGAGAGAGAGAGAGAGAGAGAGAGACACACACACAC
ACACACACACACTGTGTGTGTGTGTGTGTGTGTGTGTGTG	TGTGTGTGTGTGTGTGTGTGTGTGTGTGACACACACACAC
ACACACACACACTCTCTCTCTCTCTCTCTCTCTCTCTCTC	TCTCTCTCTCTCTCTCTCTCTCTCTCTCACACACACACAC
ACACACACACACGGGGGGGGGGGGGGGGGGGGGGGGGGGG	GGGGGGGGGGGGGGGGGGGGGGGGGGGGACACACACACAC
ACACACACACACTATATATATATATATATATATATATATA	TATATATATATATATATATATATATATAACACACACACAC
ACACACACACACATATATATATATATATATATATATATAT	ATATATATATATATATATATATATATATACACACACACAC
ACACACACACACCTCTCTCTCTCTCTCTCTCTCTCTCTCT	CTCTCTCTCTCTCTCTCTCTCTCTCTCTACACACACACAC
ACACACACACACCGCGCGCGCGCGCGCGCGCGCGCGCGCG	CGCGCGCGCGCGCGCGCGCGCGCGCGCGACACACACACAC
ACACACACACACTTTTTTTTTTTTTTTTTTTTTTTTTTTT	TTTTTTTTTTTTTTTTTTTTTTTTTTTTACACACACACAC
ACACACACACACCACACACACACACACACACACACACACA	CACACACACACACACACACACACACACAACACACACACAC
ACACACACACACACACACACACACACACACACACACACAC	ACACACACACACGCGCGCGCGCGCGCGCGCGCGCGCGCGC
GCGCGCGCGCGCGCGCGCGCGCGCGCGCACACACACACAC	ACACACACACACGTGTGTGTGTGTGTGTGTGTGTGTGTGT
GTGTGTGTGTGTGTGTGTGTGTGTGTGTACACACACACAC	ACACACACACACCCCCCCCCCCCCCCCCCCCCCCCCCCCC
CCCCCCCCCCCCCCCCCCCCCCCCCCCCACACACACACAC	GCGCGCGCGCGCGAGAGAGAGAGAGAGAGAGAGAGAGAGA
GAGAGAGAGAGAGAGAGAGAGAGAGAGAGCGCGCGCGCGC	GCGCGCGCGCGCAGAGAGAGAGAGAGAGAGAGAGAGAGAG
AGAGAGAGAGAGAGAGAGAGAGAGAGAGGCGCGCGCGCGC	GCGCGCGCGCGCTGTGTGTGTGTGTGTGTGTGTGTGTGTG
TGTGTGTGTGTGTGTGTGTGTGTGTGTGGCGCGCGCGCGC	GCGCGCGCGCGCTCTCTCTCTCTCTCTCTCTCTCTCTCTC
TCTCTCTCTCTCTCTCTCTCTCTCTCTCGCGCGCGCGCGC	GCGCGCGCGCGCGGGGGGGGGGGGGGGGGGGGGGGGGGGG
GGGGGGGGGGGGGGGGGGGGGGGGGGGGGCGCGCGCGCGC	GCGCGCGCGCGCTATATATATATATATATATATATATATA
TATATATATATATATATATATATATATAGCGCGCGCGCGC	GCGCGCGCGCGCATATATATATATATATATATATATATAT
ATATATATATATATATATATATATATATGCGCGCGCGCGC	GCGCGCGCGCGCCTCTCTCTCTCTCTCTCTCTCTCTCTCT
CTCTCTCTCTCTCTCTCTCTCTCTCTCTGCGCGCGCGCGC	GCGCGCGCGCGCCGCGCGCGCGCGCGCGCGCGCGCGCGCG
CGCGCGCGCGCGCGCGCGCGCGCGCGCGGCGCGCGCGCGC	GCGCGCGCGCGCTTTTTTTTTTTTTTTTTTTTTTTTTTTT
TTTTTTTTTTTTTTTTTTTTTTTTTTTTGCGCGCGCGCGC	GCGCGCGCGCGCCACACACACACACACACACACACACACA
CACACACACACACACACACACACACACAGCGCGCGCGCGC	GCGCGCGCGCGCACACACACACACACACACACACACACAC
ACACACACACACACACACACACACACACGCGCGCGCGCGC	GCGCGCGCGCGCGCGCGCGCGCGCGCGCGCGCGCGCGCGC
GCGCGCGCGCGCGTGTGTGTGTGTGTGTGTGTGTGTGTGT	GTGTGTGTGTGTGTGTGTGTGTGTGTGTGCGCGCGCGCGC
GCGCGCGCGCGCCCCCCCCCCCCCCCCCCCCCCCCCCCCC	CCCCCCCCCCCCCCCCCCCCCCCCCCCCGCGCGCGCGCGC
GTGTGTGTGTGTGAGAGAGAGAGAGAGAGAGAGAGAGAGA	GAGAGAGAGAGAGAGAGAGAGAGAGAGAGTGTGTGTGTGT
GTGTGTGTGTGTAGAGAGAGAGAGAGAGAGAGAGAGAGAG	AGAGAGAGAGAGAGAGAGAGAGAGAGAGGTGTGTGTGTGT
GTGTGTGTGTGTTGTGTGTGTGTGTGTGTGTGTGTGTGTG	TGTGTGTGTGTGTGTGTGTGTGTGTGTGGTGTGTGTGTGT
GTGTGTGTGTGTTCTCTCTCTCTCTCTCTCTCTCTCTCTC	TCTCTCTCTCTCTCTCTCTCTCTCTCTCGTGTGTGTGTGT
GTGTGTGTGTGTGGGGGGGGGGGGGGGGGGGGGGGGGGGG	GGGGGGGGGGGGGGGGGGGGGGGGGGGGGTGTGTGTGTGT
GTGTGTGTGTGTTATATATATATATATATATATATATATA	TATATATATATATATATATATATATATAGTGTGTGTGTGT
GTGTGTGTGTGTATATATATATATATATATATATATATAT	ATATATATATATATATATATATATATATGTGTGTGTGTGT
GTGTGTGTGTGTCTCTCTCTCTCTCTCTCTCTCTCTCTCT	CTCTCTCTCTCTCTCTCTCTCTCTCTCTGTGTGTGTGTGT
GTGTGTGTGTGTCGCGCGCGCGCGCGCGCGCGCGCGCGCG	CGCGCGCGCGCGCGCGCGCGCGCGCGCGGTGTGTGTGTGT
GTGTGTGTGTGTTTTTTTTTTTTTTTTTTTTTTTTTTTTT	TTTTTTTTTTTTTTTTTTTTTTTTTTTTGTGTGTGTGTGT
GTGTGTGTGTGTCACACACACACACACACACACACACACA	CACACACACACACACACACACACACACAGTGTGTGTGTGT
GTGTGTGTGTGTACACACACACACACACACACACACACAC	ACACACACACACACACACACACACACACGTGTGTGTGTGT
GTGTGTGTGTGTGCGCGCGCGCGCGCGCGCGCGCGCGCGC	GCGCGCGCGCGCGCGCGCGCGCGCGCGCGTGTGTGTGTGT
GTGTGTGTGTGTGTGTGTGTGTGTGTGTGTGTGTGTGTGT	GTGTGTGTGTGTCCCCCCCCCCCCCCCCCCCCCCCCCCCC
CCCCCCCCCCCCCCCCCCCCCCCCCCCCGTGTGTGTGTGT	CCCCCCCCCCCCGAGAGAGAGAGAGAGAGAGAGAGAGAGA
GAGAGAGAGAGAGAGAGAGAGAGAGAGACCCCCCCCCCCC	CCCCCCCCCCCCAGAGAGAGAGAGAGAGAGAGAGAGAGAG
AGAGAGAGAGAGAGAGAGAGAGAGAGAGCCCCCCCCCCCC	CCCCCCCCCCCCTGTGTGTGTGTGTGTGTGTGTGTGTGTG
TGTGTGTGTGTGTGTGTGTGTGTGTGTGCCCCCCCCCCCC	CCCCCCCCCCCCTCTCTCTCTCTCTCTCTCTCTCTCTCTC
TCTCTCTCTCTCTCTCTCTCTCTCTCTCCCCCCCCCCCCC	CCCCCCCCCCCCGGGGGGGGGGGGGGGGGGGGGGGGGGGG
GGGGGGGGGGGGGGGGGGGGGGGGGGGGCCCCCCCCCCCC	CCCCCCCCCCCCTATATATATATATATATATATATATATA
TATATATATATATATATATATATATATACCCCCCCCCCCC	CCCCCCCCCCCCATATATATATATATATATATATATATAT
ATATATATATATATATATATATATATATCCCCCCCCCCCC	CCCCCCCCCCCCCTCTCTCTCTCTCTCTCTCTCTCTCTCT
CTCTCTCTCTCTCTCTCTCTCTCTCTCTCCCCCCCCCCCC	CCCCCCCCCCCCCGCGCGCGCGCGCGCGCGCGCGCGCGCG
CGCGCGCGCGCGCGCGCGCGCGCGCGCGCCCCCCCCCCCC	CCCCCCCCCCCCTTTTTTTTTTTTTTTTTTTTTTTTTTTT
TTTTTTTTTTTTTTTTTTTTTTTTTTTTCCCCCCCCCCCC	CCCCCCCCCCCCCACACACACACACACACACACACACACA
CACACACACACACACACACACACACACACCCCCCCCCCCC	CCCCCCCCCCCCACACACACACACACACACACACACACAC
ACACACACACACACACACACACACACACCCCCCCCCCCCC	CCCCCCCCCCCCGCGCGCGCGCGCGCGCGCGCGCGCGCGC
GCGCGCGCGCGCGCGCGCGCGCGCGCGCCCCCCCCCCCCC	CCCCCCCCCCCCGTGTGTGTGTGTGTGTGTGTGTGTGTGT
GTGTGTGTGTGTGTGTGTGTGTGTGTGTCCCCCCCCCCCC	CCCCCCCCCCCCCCCCCCCCCCCCCCCCCCCCCCCCCCCC

## Data Availability

The original contributions presented in this study are included in the article. Further inquiries can be directed to the corresponding author.
